# Striking a balance: how the gut microbiome shapes the fate of intestinal CD4+ T cells

**DOI:** 10.1093/discim/kyaf020

**Published:** 2025-12-09

**Authors:** Jessica M Till, Orion D Brock, Philip P Ahern

**Affiliations:** Department of Cardiovascular and Metabolic Sciences, Cleveland Clinic Research, Cleveland Clinic, Cleveland, OH 44195, USA; Center for Microbiome and Human Health, Cleveland Clinic Research, Cleveland Clinic, Cleveland, OH 44195, USA; Department of Molecular Medicine, Cleveland Clinic Lerner College of Medicine, Case Western Reserve University, Cleveland, OH, USA; Department of Cardiovascular and Metabolic Sciences, Cleveland Clinic Research, Cleveland Clinic, Cleveland, OH 44195, USA; Center for Microbiome and Human Health, Cleveland Clinic Research, Cleveland Clinic, Cleveland, OH 44195, USA; Department of Molecular Medicine, Cleveland Clinic Lerner College of Medicine, Case Western Reserve University, Cleveland, OH, USA; Department of Cardiovascular and Metabolic Sciences, Cleveland Clinic Research, Cleveland Clinic, Cleveland, OH 44195, USA; Center for Microbiome and Human Health, Cleveland Clinic Research, Cleveland Clinic, Cleveland, OH 44195, USA; Department of Molecular Medicine, Cleveland Clinic Lerner College of Medicine, Case Western Reserve University, Cleveland, OH, USA

**Keywords:** gut microbiome, CD4+ T cells, immune tolerance, intestine, mucosal immunology

## Abstract

The induction of immune tolerance, a state of immunologic hyporesponsiveness to an antigen, is essential to prevent the destructive potential of the immune system in response to harmless or beneficial agents. Early efforts to understand tolerance focused on model stimuli, self-antigens, transplanted organs, and the growing fetus. Through co-evolution, the microbiome and the host immune system have developed strategies that promote immunological tolerance to the microbiome. This dialogue ensures the maintenance of mutualistic interactions that provide a stable habitat for the microbiome which in turn confers numerous physiological benefits to the host. Despite the gut microbiome being a potent inducer of immune tolerance, the mechanisms through which specific members shaped immune function remained largely ignored for decades. The growing appreciation for the immunomodulatory capacity of the microbiome has led to a massive expansion of efforts to define how the balance between tolerance and inflammation is induced and maintained at mucosal sites like the intestine. While the ensuing research uncovered myriad fundamental insights into the concerted host and microbial functions promoting host-microbiome mutualism, inducing tolerance to clinically relevant antigens remains a major challenge in the development of tolerogenic therapies. Here, we trace the interaction between intestinal CD4+ T cells and the microbiome, from antigen uptake through to the development of a polarized collection of CD4+ T cells, whose functions are essential for immunological tolerance, and highlight the knowledge gaps that limit efforts to leverage these interactions for clinical benefit.

## Introduction

Foundational studies describing the generation of immunological tolerance by Owen, Burnet, Medawar and others inspired immunologists to understand the mechanistic underpinnings of this phenomenon, in hopes of gaining insights into the etiology of autoimmune disease and providing novel avenues for the treatment of immune-driven disorders [1–5]. Nowhere are the challenges associated with the induction of tolerance more evident than the intestine, a unique immunologic site home to a vast collection of bacterial, fungal, viral, and archaeal agents, collectively referred to as the microbiome, as well as a dense network of immune cells. Given the potential for these immune cells to generate tissue-destructive responses in response to stimulation with microbial products, it is essential that the microbes (used here to include viruses) that make up the microbiome and the intestinal immune system engage in harmonious interactions. Despite the significance of these relationships for host health, the dynamic immune operations that sustain mutualistic interactions at intestinal mucosal sites were long studied only by a small cadre of researchers, and this “frontier of the immune system” [6, 7] long remained the “poor relation of the immunology world” [8]. A rapid increase in our understanding of the composition and dynamic operations of the microbiome [9–12], coupled with striking examples of how the microbiome shaped intestinal immunity [13–18], kindled interest in identifying the most potently immunomodulatory microbiome members, and defining the mechanisms that underlie these interactions.

The inflammatory potential of the gut microbiome necessitates adaptations that prevent unfettered access to host tissues and their embedded immune systems, as well as immunologic adaptations that prevent deleterious responses following exposure to microbiome-derived products from beneficial or harmless microbes. The intestinal epithelium and its overlying mucus layer provide an effective physical barrier that limits exposure of the underlying intestinal immune system to the products from the gut microbiome. The critical nature of this physical barrier is exemplified by the deleterious impacts of impaired epithelial barrier function on host health. Despite its effectiveness, immune cells in the intestine, mesenteric lymph nodes (MLN), and gut-associated lymphoid tissues (GALT) are continuously being exposed to immune stimulatory products and foreign antigens (defined here as antigens not encoded in the host genome) [19, 20]. Upon encounter with these factors, the intestinal immune system must respond in a manner appropriate to the nature of the challenge. As such, the immune system functions as a sophisticated decision-making device that integrates quantitative and qualitative features of its environment to mount a response that is tailored to effectively manage the various entities it encounters. Making the “correct” decision is critical as inappropriate inflammatory responses to harmless/beneficial microbiome members promote tissue destruction, while tolerogenic responses that limit the capacity to restrain harmful pathogens can lead to severe illness or death. Despite these challenges, the immune system reliably generates appropriate responses, effectively balancing protective immunity against pathogens while maintaining mutualistic interactions with the microbiome.

How the immune system achieves this balance has intrigued immunologists for the past few decades and prompted efforts to define the molecular and cellular mechanisms through which microbiome members elicit tolerogenic host immune responses despite exposure to immune-stimulatory compounds and non-self-antigens from both symbiotic and pathogenic bacteria. Ultimately, intestinal immune tolerance is shaped by the cooperative function of both immune and non-immune cells in response to microbial signals derived from the microbiome. Central in this intricate network are CD4+ T cells, which facilitate the coordination of finely tuned and highly microbe-specific responses to neutralize threats or maintain intestinal homeostasis. Although the study of immune tolerance in the intestine has a rich history built on the investigation of tolerance to orally administered antigens [8, 21], the capacity of gut microbes to induce immune tolerance has received considerably less attention until recently. Here, we review the current understanding of how intestinal CD4+ T cell responses to the gut microbiome are generated, tracing the immune response from the initial acquisition of antigen to the final accumulation of polarized effector CD4+ T cells in the gut, and highlight the many gaps which still remain in our understanding of the intricate networks involved. Many outstanding studies regarding the impact of the microbiome on other key cell types, body sites, and temporal aspects of these responses are not discussed here due to space constraints, and we recommend to the reader several outstanding reviews that address these interactions [22–25], thus providing a comprehensive understanding of immune microbiome relationships.

## CD4+ T cell responses to the microbiome

CD4+ T cell activation is driven by recognition of antigens presented on major histocompatibility complex (MHC) II by antigen presenting cells (APC), where they can be recognized by the T cell receptor, in conjunction with costimulatory signals from the APC [26]. One of the central features of CD4+ T cells is their capacity to adopt a specific cellular fate depending on the nature of the stimulus to which it reacts. As such, CD4+ T cells can adopt a variety of fates, including T helper (Th)1, Th2, Th17, Th9, regulatory T cell (Treg), and T follicular helper (Tfh) that are characterized by expression of particular transcription factors required to program stereotypical patterns of cytokine secretion. Although it is clear that these fates exist on a continuum [27–29], this framework has proved a useful means to conceptualize how CD4+ T cell function is shaped in response to microorganisms [26]. Unsurprisingly, there exists a close relationship between the gut microbiome and CD4+ T cell phenotype and function in the intestine [30]. Notably, variation in microbiome composition contributes to variance in intestinal T cell phenotypes, and as such, microbiome composition represents a driver of inter-individual intestinal CD4+ T cell phenotypes [30]. Mounting an appropriate response to the microbiome is essential, as dysfunctional immune-microbiome interactions can precipitate autoimmune and inflammatory disorders. Thus, defining the molecular pathways that govern T cell responses to the microbiome is critical for understanding intestinal immune tolerance, and offers an opportunity to derive insights into how the immune system can be programed to promote tolerance to foreign entities.

At steady state, the intestinal lamina propria CD4+ T cell compartment is dominated by FoxP3+ Tregs [13, 14, 31–33] and Retinoic Acid Receptor–related Orphan Receptor γt (RORγt+) Th17 cells [13, 15–17, 34] which accumulate in a microbiome-dependent manner. Although the anti-inflammatory functions of Tregs are fundamental to our symbiotic partnerships [35], it is increasingly evident that microbiome-induced Th17 cells can contribute to the quiescent immune-microbiome state through their anti-inflammatory [36] and anti-microbial functions [37], their inflammatory potential notwithstanding [38, 39]. Although tolerance was originally described as “a state of indifference or non-reactivity toward a substance that would normally be expected to excite an immunological response” [40], it is now more commonly conceptualized to include the role played by immune cells, such as Tregs, in actively maintaining a non-inflammatory state through potent anti-inflammatory functions in response to “substances.” Here, we will consider the role played by Th17 and Tfh cells during steady state conditions in promoting immune ignorance through anti-microbial function and antibody induction, respectively, in keeping with the originally envisioned meaning of the term tolerance, and representing a critical arm that ensures the maintenance of mutualistic responses. However, the potent inflammatory potential of intestinal Th17 cells [38, 39], which distinguishes them from Tregs whose “raison d'être” is to limit inflammatory responses, is critical when considering their role as mediators of “tolerance.”

**Figure 1:**
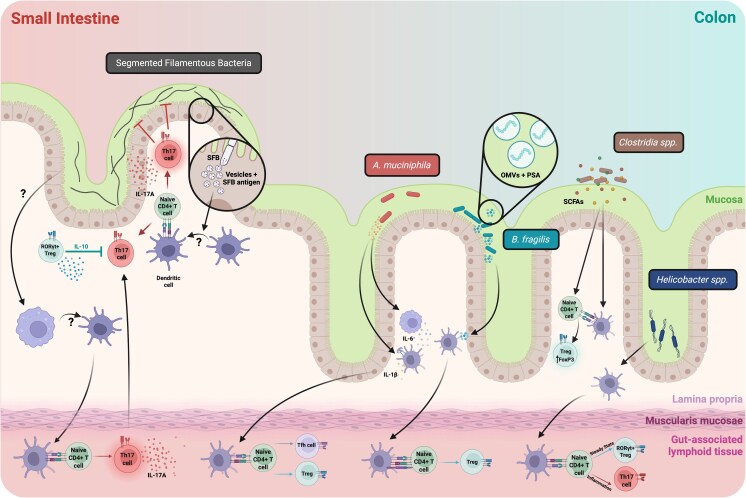
Schematic of mechanisms of bacterial colonization, antigen acquisition, and presentation to CD4+ T cells in the intestine. Created in BioRender. Engelhart, M. (2025) https://BioRender.com/s6vv57i.

The microbiome-dependent accumulation of intestinal Treg and Th17 cells prompted intense investigation to identify the microbiome members responsible. Intestinal Tregs are potently immunosuppressive and express a suite of anti-inflammatory molecules, including interleukin-10 (IL-10), that prevent inflammatory responses directed against the microbiome and host tissue [35]. Treg induction has been shown to be a property of several different bacteria including a consortia of *Clostridia* strains [13, 14], the Altered Schaedler Flora (ASF) [31], several members of the genus *Bacteroides* [32, 33, 41, 42], various *Helicobacter* species [43–46], as well as *Bifidobacteria* [47]. Additionally, FoxP3- IL-10-producing T regulatory 1 (Tr1) cells can be induced by *Bifidobacterium breve* [48] (Fig. 1). While the intestine is home to a population of Tregs that develop in the thymus (thymic Treg; tTreg) and can be found in germ-free mice, a large proportion of colonic Tregs are microbiome-elicited cells that are posited to arise in the periphery from naïve precursors (peripheral Treg; pTreg), although formal demonstration of peripheral differentiation has only been obtained for a handful of microbes. Although no perfect marker for pTregs exists, colonic Tregs are enriched for cells that express the transcription factor RORγt in conjunction with FoxP3 [33, 49, 50]. Prior to the identification of RORγt as a pTreg marker, earlier studies utilized expression of HELIOS [51] and Neuropilin-1 [52, 53], as low or no expression of these markers had been linked to pTreg development using transgenic TCR expressing T cells and these cells were largely absent from germ-free mice. However, the use of HELIOS and Neuropilin-1 as definitive markers of pTregs has since been challenged [54–56]. While cell transfer studies support the concept of RORγt marking cells that arise from naïve precursors in the periphery, important limitations also exist [57–59], and it seems likely that no marker that can truly differentiate between pTreg and tTreg exists. RORγt expression in intestinal Tregs is microbiome-dependent, and microbiome-specific intestinal Tregs are typically uniformly RORγt+; however, it remains to be addressed whether all RORγt+ Tregs are microbiome-reactive. Moreover, as RORγt-FoxP3+ cells can proceed to being RORγt+FoxP3+ [60], it is possible that self-specific cells that develop intra-thymically as RORγt-FoxP3+ may also form part of the RORγt+ Treg pool in response to microbial stimulation, confounding the utility of RORγt as a marker of pTreg. pTregs play a critical role in limiting intestinal inflammation, with deletion of RORγt within the FoxP3+ compartment leading to development of more severe intestinal inflammation [33, 50, 61], likely attributable to declining FoxP3 expression in RORγt-deficient Tregs [61]. While these studies suggest that pTregs play a critical role in limiting pro-inflammatory responses in the gut, it is unclear if this is due to the loss of Tregs specific for gut microbiome members or some facet of RORγt function itself that drives tolerogenic programs in response to the microbiome. These findings have been interpreted to demonstrate a unique requirement for RORγt+ Treg at mucosal surfaces, however, it is likely that intestinal homeostasis is achieved by the dual activity of tTregs and pTregs as has been demonstrated for the prevention of colitis in the T cell transfer model [62]. To date, a lack of tools for the specific depletion of tTregs has limited further insights into their contribution to intestinal homeostasis.

The induction of Th17 responses is somewhat more selective and was initially thought to be unique to segmented filamentous bacteria (SFB) colonization [15–17]. Now, a range of microbes, as well as the parasite *Tritrichomonas musculis*, have been identified to have Th17 inducing capacity [13, 34, 63]. While Th17 cells are understood to develop in the periphery, the microbiome can impact the thymic selection of SFB-specific αβ T cells [64]. Interestingly, microbiome-elicited Th17 cells are typically more dominant in the small intestinal lamina propria [16, 17, 34], while microbiome-elicited Tregs accumulate in the colon [13, 14, 16, 17, 31–34]. It is unknown whether site specific T cell polarization is a feature of microbe localization or a form of tissue control [67] whereby the fate of microbiome-reactive cells is imprinted by local tissue factors such that an optimal response for that specific tissue is generated.

Despite the pathogenic potential of Th17 cells in other tissues, microbiome-elicited Th17 cells are typically not inflammatory and benefit the host through their impact on microbiome composition [37], improvement of intestinal barrier function [37], and providing pre-existing immunity to combat pathogens [16]. For example, SFB-induced intestinal Th17 cells contribute to intestinal homeostasis by limiting the expansion of SFB itself, which can elicit pathogenic responses when its abundance is not contained [37. Furthermore, SFB induces a self-restraining anti-inflammatory feedback loop that includes the production of IL-10, that limits the pathogenic function of SFB-induced Th17 cells [36]. Both Tregs and Th17 cells further contribute to immune homeostasis through expression of a tissue reparative program characterized by expression of amphiregulin that likely helps to ensure tissue integrity, as at other body sites [68, 69], although amphiregulin has been linked to the development of fibrosis [70, 71]. The transcription factor c-MAF is critical for the development of these programs in both Tregs [45] and Th17 cells [36] and the collective actions of these cells serves to dampen inflammation through distinct and overlapping mechanisms to maintain immune-microbiome mutualism.

Besides Tregs and Th17 cells, the promotion of intestinal Tfh responses by the microbiome has been recently uncovered and appears to represent a cell fate induced by many gut bacteria [45, 72]. Interestingly, when *Akkermansia muciniphila* is colonized in mice together with the ASF, a Tfh program is the dominant fate adopted by *A. muciniphila*-specific CD4+ T cells, but in the context of a more complex microbiome, *A. muciniphila*-specific Treg, Th17 and Th1 cells emerge in addition to Tfh cells, and thus microbiome variation may shape the output of CD4+ T cells *in trans* [22]. Although the role of these microbiome-specific Tfh cells has not been extensively explored, it is likely that they promote the generation of high-affinity immunoglobulin (Ig) A antibodies that target the microbe for which these T cells are specific. Intestinal IgA plays a fundamental role in promoting host-microbiome mutualism [23, 73, 74] and is thought to preferentially coat immunogenic microbes to prevent penetration into host tissues and neutralize microbially derived inflammatory products [75, 76]. Microbiome-specific T-independent IgA exists [77], suggesting that intestinal T cells are not required for the generation of microbiome-specific IgA but instead form a feedback loop whereby IgA is induced by select microbes to self-regulate gut microbiome composition [78, 79]. Thus, microbiome-driven modulation of intestinal T cell fate can contribute to tolerance through modulation of IgA responses. Strikingly, although Tregs [80] and Th17 cells [81] have also been linked to induction of class switching to IgA, several reports show that Th17 cells and Tregs can adopt a Tfh fate as a means to shape intestinal antibody production [82, 83]. Although a more comprehensive review of the impact of the microbiome on intestinal antibodies is beyond the scope of this review, this topic has been extensively reviewed elsewhere [23, 73, 84].

In summary, the collective activity of intestinal CD4+ T cells sustains mutualistic interactions with the gut microbiome. This can be mediated through active inhibition of inflammatory pathways by Treg cells, through their production of cytokines like IL-10, but additionally through Th17 cells which promote immune ignorance by limiting the exposure if the host to various microbial products, both through promoting intestinal barrier function, anti-microbial function, as well as limiting host penetration by the microbiome through the generation of microbiome-specific antibodies. However, it remains to be fully understood how all of these functions are coordinated.

## First encounters

The first step in priming CD4+ T cell responses involves the acquisition of antigen that must be presented to naïve CD4+ T cells by antigen-presenting cells (APC). Orally administered model antigens have been extensively used to define the uptake of antigen in the intestine [85]. However, we still have a limited understanding of the form that microbial-derived antigens are first encountered and if the same pathways mediate microbial and dietary antigen uptake.

Several means of antigen acquisition from the intestine have been uncovered using orally delivered free antigen. Microfold (M) cells which overlie the follicular associated epithelium (FAE) of GALT represent one of the best described processes. M cells are largely devoid of the thick overlying mucus layer that characterizes the intestine, which is believed to allow them to be efficient points of antigen acquisition and both dendritic cells (DC) and B cells (through a DC-independent process) can capture antigen from/through M cells [86–88. The availability of M cell deficient mice has allowed assessment of the requirement for these cells in antigen uptake, and revealed a role for M cells in the generation of Tfh cells and subsequent IgA in the Peyer’s patches (PP) [89]. However, despite their clear role in mounting immunity to pathogens [85, 90], there is a paucity of information regarding their role in uptake of specific microbiome members, or if their capacity to respond differently to distinct bacteria [91] plays a role in conditioning the intestinal T cell response. M cells are prevalent in the PP of the small intestine, however, the colon is the site of the densest colonization by the microbiome but lacks PPs. Instead, the colon harbors a caecal patch and colonic patch which represent induction sites for B and T cell responses [92, 93]. Moreover, solitary isolated lymphoid tissues (SILTs), including both cryptopatches (CP) and isolated lymphoid follicles (ILF), are distributed along the length of the small intestine and colon in both mice and humans, representing alternative induction sites to the PPs [94, 95]. Given that they also possess M cells [96, 97], ILFs are likely an important site of induction of adaptive immune responses in the colon, functioning akin to PPs [97, 98]. Moreover, ILFs are microbiome-responsive [99], and bacterial species such as SFB can induce the development of secondary lymphoid follicles that act as induction sites independently of PPs [100]. Extensive variation between individual ILFs in the small intestine suggests they likely function as unique inductive sites, tailoring to the needs of individual regions of the intestine [101]. To date little is known about the requirement for these structures in the coordination of responses to the microbiome, but the distinct development requirements in mice between small intestine and colon ILFs may afford the opportunity to tease apart their role in mediating responses to the microbiome [102, 103]. Difficulties with the removal of ILF structures prior to phenotyping of intestinal tissue, allied to challenges associated with the genetic depletion of ILFs, has made it challenging to uncover their contributions to intestinal T cell function [104, 105]. However, elegant studies in humans where these structures can be visualized and removed have led to the identification of two types of ILFs, mucosal-ILF and submucosal-ILF, that differ in their location within the tissue and their cellular composition [105]. Thus, ILFs located at different sites along and within the intestine likely provide unique functions. While the presence of naïve T cells in these structures suggests that they may represent a site at which microbiome-specific cells are primed [105, 106], further studies are required to delineate the contributions of these distinct sites to the polarization of microbiome-specific T cells.

As an alternative to M cell uptake in GALT, it has been posited that some APCs acquire luminal antigen by extending long cellular protrusions, or transepithelial dendrites (TED), through the epithelial layer [107–110]. While this process has been visualized via two-photon microscopy, the lack of tools that would allow for deletion of this function has meant that it is difficult to assess the relative contribution of this process to antigen acquisition. More recently, mucus-secreting goblet cells have been demonstrated to possess antigen uptake capabilities [111, 112]. These cells readily take up antigen and particles through goblet cell-associated antigen passages (GAP) via endocytosis and deliver the antigen to APCs via transcytosis [113]. Depletion of goblet cells using *Math1*^fl/fl^ X *Vil*^Cre-ERT2^ mice or inhibition of GAP formation through provision of luminal murine epidermal growth factor which inhibits GAP formation through the epidermal growth factor receptor (EGFR), reveals that GAP structures and goblet cells are required for the transfer of orally provided ovalbumin to APCs, and the optimal induction of anti-inflammatory pTregs in the small intestine [111, 114]. During intestinal *Salmonella* infection, GAP formation is inhibited, limiting the proliferation of CD4+ T cells specific for dietary antigen. This inhibition of GAP formation likely sustains tolerance to dietary antigens during infection by limiting their uptake together with pathogens that concurrently are driving a proinflammatory response [115]. Despite their prominent role in mediating immunity to dietary antigen, mice with impaired GAPs still harbor robust populations of RORγt+ Tregs in the colon [111] suggesting that they are not required for induction of microbiome-specific Tregs at this site. Indeed, MyD88-dependent goblet cell-sensing of microbes actively limits colonic GAP formation to prevent inordinate inflammatory responses to the microbiome [114]. Therefore, it is possible that both the site of uptake and the source of the antigen may impact the route(s) of antigen acquisition, with the induction of tolerance to dietary antigens operating through distinct pathways to that of microbially-derived antigens. Much work remains to be done to track the pathways and forms through which microbiome-derived antigens are transferred to APC in the process of initiating microbiome-specific CD4+ T cell responses.

A growing body of work suggests that outer membrane vesicles (OMV) from Gram negative bacteria [116] and membrane vesicles (MV) from Gram positive bacteria and fungi [117] harbor immunomodulatory potential [118–123], and represent a strategy that allows the microbiome to intentionally shape intestinal immune responses. These vesicles can cross the epithelium [118, 119] and carry antigens that are recognized by microbiome-specific CD4+ T cells [42, 124], suggesting that the antigen acquisition by APC is through uptake of such vesicles rather than free antigen. Cell wall products can also cross the epithelium in vesicles or in vesicle-free form [119, 125] and may provide a source of factors that condition the response through stimulation of pattern recognition receptors (PRRs). Moreover, live bacteria can translocate to the MLN, potentially within live DCs, under homeostatic conditions [113, 126]. However, a dearth of tools that can precisely inhibit any of these processes within the bacteria themselves has limited our ability to determine which of these processes is most important for the stimulation of CD4+ T cell responses. Recent advances identifying signals that govern the localization of factors to such vesicles may allow assessment of this question.

The localization of microbes within the gut lumen or the mucosal interface has long been posited as an important factor governing their immunomodulatory potential. SFB, which represents one of the most immunogenic gut microbes identified to date [15, 16, 18, 127, 128], penetrates the intestinal mucosa and forms close associations with the host epithelium [129–131], possibly through the abundant glycosyl hydrolases and glycan utilization genes in its genome [132]. SFB adheres to intestinal barrier cells via use of a holdfast structure that attaches to the cell plasma membrane without rupturing the cell [124, 133, 134], and this attachment is thought to be critical for its immunomodulatory potential. Specifically, mouse-derived SFB potently induces Th17 cells in mice but not in rats, while rat-derived SFB potently induces Th17 cells in rats but not in mice [135]. Although both isolates show efficient cross-species colonization, mouse-derived SFB does not adhere to rat epithelium and vice versa [135, 136], thus supporting the concept that SFB induces Th17 accumulation only in hosts in which it adheres to the epithelium. Interestingly, SFB-specific Th17 cells are most enriched in the ileum, the site with most abundant SFB adherence, further corroborating the role of adherence in this response, possibly though increasing antigen availability [16, 135, 137]. SFB directly transmits vesicles containing CD4+ T cell antigens to the host via adherence-triggered endocytosis, and interference with these endocytic events through deletion of epithelial cell CDC42 impairs the ability of SFB to induce Th17 cells, despite normal attachment to intestinal epithelial cells (IEC) [124, 129]. Thus, Th17 induction is not mediated by epithelial adherence alone, but by the downstream transfer of SFB-derived material across the epithelium. While we lack similar levels of mechanistic insight for other microbiome members, *Clostridia* spp. [13, 14, 128, 138]*, Helicobacter* spp. [44–46], *Bacteroides fragilis* [41], and *A. muciniphila* [72] are all potent modulators of intestinal T cell fate in steady state conditions and can reside in close contact with the host by colonizing the colonic crypts or the mucosa, suggesting that intimate contact with host epithelial cells at this location is important for immunomodulatory capacity. Although obtaining formal proof of this concept has been challenging, deletion of a *BF3134*-encoded glycosyl hydrolase in *B. fragilis* impairs mucus colonization, leading to a reduced induction of IL-10-producing Tregs [139, further suggesting that intimate proximity and contact with host epithelial cells is important for immunomodulatory capacity [72, 140–144].

Despite progress in our understanding of antigen acquisition by the intestinal immune system, the nature of the initial uptake of microbial antigens in the gut remains one of the most poorly understood aspects of immune interaction with the microbiome. Consequently, we have little information as to whether the mode of acquisition impacts the fate of CD4+ T cells or simply represents a highly redundant step to pass antigens to APCs for the activation and differentiation of intestinal T cells.

## Unexpected antigen-presenting cell complexity

Following the acquisition of antigen, the next step in priming of CD4+ T cell responses to the microbiome requires antigen presentation by a “professional” APC that can activate a naïve CD4+ T cell. APCs are critical regulators of CD4+ T cell fate following activation, sensing conserved microbial products through PRRs in order to provide distinct T cell polarizing signals that elicit the differentiation of the appropriate cell subset [26]. APCs thereby link microbial features with specific antigens to help ensure a response that both coordinates appropriate downstream immune functions and is specific to the eliciting agent. The superior capacity of conventional/classical dendritic cells (cDC) relative to other APCs to migrate from sites of antigen uptake to draining lymph nodes where a large pool of naïve CD4+ T cells are available for activation, identified them as the most likely cell type for priming microbiome-specific T cell responses [145–147]. Intestinal cDCs are CD11c+MHCII+CD64- cells dominated by three predominant subsets: CD103+CD11b-(cDC1), CD103+CD11b+ (cDC2), and CD103-CD11b+ (cDC2) [148, 149]. Elegant *ex vivo* assays demonstrate that these DCs are the predominant capturer of intestinal antigen for priming in the MLN, endowed with the capacity to migrate to the MLN and promote Treg [150, 151] and Th17 [152] differentiation under steady state conditions. Various genetic systems that facilitate the depletion of individual cDC subsets further suggested a distinct role for different subsets of DCs in the priming of Treg or Th17 responses [153–156]. However, more recent efforts using newer tools to delineate the precise antigen presenting cells that promote the development of microbiome-specific T cells has uncovered previously unappreciated complexity.

Examination of the APC requirements for the induction of SFB-specific Th17 responses revealed that MHCII expression by CD11c+ cells is both essential and sufficient [66], as would be expected for a cDC-driven event, and was consistent with the reduced intestinal Th17 accumulation reported in mice lacking CD103+CD11b+cDC2 [153–156]. However, this was subsequently posited to be due to CD11c+ macrophages as “FMS-like tyrosine kinase 3 ligand” (*Flt3l*)*^-/-^* mice that have severely reduced cDCs [157] showed unimpaired generation of SFB-driven Th17 cells [158]. Furthermore, C-C chemokine receptor 2 (CCR2)-mediated depletion of macrophages, or anti-colony stimulating factor (CSF)1-mediated inhibition of macrophage development, significantly reduced the accumulation of SFB-driven Th17 cells, while monocyte transfer corrected this defect, thus collectively demonstrating a requirement for macrophages in the response. Importantly, SFB-induced Th17 induction can occur in the absence of all known secondary lymphoid tissues, including MLNs, in lymphotoxin A (LTα)-deficient mice [65, 66, 159], thereby providing a plausible mechanism by which macrophages, which do not migrate to the MLNs [160], could mediate SFB-specific Th17 cell induction. This is in contrast to other studies which suggest that MLNs are the site of SFB-specific Th17 cell induction under normal conditions [161, 162]. Furthermore, a separate study that used a combination of C-C chemokine receptor 7 (CCR7)-deficiency and depletion of cDCs using a ZBTB46-diphtheria toxin receptor (DTR) system where diphtheria toxin (DT) administration ablates cDCs [163], suggested that cDC migration to the MLN is required for SFB-specific Th17 cell induction [162]. While the reason for this discrepancy is unclear, it is known that although substantially impaired, *Flt3l^-/-^* mice develop a limited number of cDCs [157], and it is possible that these cells are absent in ZBTB46-DTR mice following DT treatment. Furthermore, while cell depletion strategies can uncover a role for a specific cell type, they do not establish them as the critical cell for presentation of antigen. As macrophages can transfer orally-fed antigen to CD103+ DCs via connexin 43 [164], the critical role for macrophages in priming of SFB-specific responses may be in the capture and transfer of SFB antigen to the migratory DCs that activate naïve T cells, but it is as yet unknown if such transfer occurs with microbiome-derived antigen. Finally, the studies showing a role for macrophages assessed Th17 cell development in response to SFB colonization in a polyclonal CD4+ T cell repertoire, while those showing a role for cDCs assessed Th17 development in a monoclonal TCR transgenic system with specificity for a single SFB-encoded antigen. Thus, it is possible that differences in the approaches used further complicate final interpretation.

Studies have also proposed that cDCs prime responses to other gut microbiome members, as cDC ablation using the ZBTB46-DTR system leads to a failure to accumulate *Helicobacter*-specific CD4+ T cells in the MLN [43]. Despite proposals that CD103+ DCs are the critical drivers of Treg development [150, 151, 156], none of the CD103+ subsets previously established to have potent Treg-inducing capacity are required for Treg generation in this system [165]. While the residual development of CD103+ DC subsets in the various genetic-depletion systems used means that a requirement for these cells cannot be unequivocally ruled out, these data suggested a highly redundant process whereby any of a variety of cDC subsets could support the development of *Helicobacter*-specific Tregs. Notably, prior work with the same *Helicobacter*-specific T cells using an alternative mechanism of depletion of CD103+CD11b+ cells via CD11c-Cre driven deletion of Notch2, did impair Treg development [43]. This discrepancy highlights how the specific depletion systems used may impact interpretation rather than simply the loss of a particular cDC subset. In a separate study of *H. hepaticus*-specific CD4+ T cells, in keeping with the concept that the MLN is the site of Treg induction in *H. hepaticus*-specific CD4+ T cells [166], CCR7-dependent migratory CD11c+MHCII+ APCs were established as the required carrier of *H. hepaticus* antigens and the most potent activator of the canonical RORγt+ Treg program, consistent with a role for cDCs in this process [167]. Intriguingly, this induction of *H. hepaticus*-specific Tregs also required expression of the transcription factor RORγt by CD11c+ cells. As RORγt is uniformly expressed by type 3 innate lymphoid cells (ILC3) which had also been established to have antigen presenting capacity [168], and were concurrently proposed to prime *H. hepaticus*-specific Tregs [169], this called into question the identity of these cell types. Are they CD11c+ ILC3s, or cDCs that express, or formerly expressed, RORγt, or are they the newly described Thetis Cells (TC) [170], or autoimmune regulator (AIRE)+ Janus Cells (JC) [171, 172]?

In support of a DC subset driving pTregs, recent studies have described PRDM16-expressing APCs that are distinct from ILC3s, and whose development is dependent on a *cis*-regulatory element 7 kb upstream from the RORγt transcription start site, as the cell type that is essential for microbiome-driven and oral antigen-driven pTreg development [173, 174]. These cells transcriptionally resemble DCs rather than ILCs and have consequently been termed tolerizing DCs (tolDCs) [173]. In keeping with the primacy of these cells for pTreg induction, their numbers remain stable through at least 12 weeks of age [173] and could explain the generation of pTregs in mice long after the weaning period [167]. By contrast, significant evidence also supports TCs as the key pTreg generating cell type [170]. MHCII deletion in cDC1s and cDC2s using Clec9a-Cre [175] or in ILC3s using RORα-Cre does not impact accumulation of colonic pTregs, suggesting that neither cell type is responsible for pTreg generation [170]. Furthermore, TCs express CD11c, ZBTB46, RORγt, and CCR7 and have variable expression of *prdm16* [170, 173, 176, 177], all of which have previously been used to identify cDCs or ILC3s as the cell type inducing microbiome or dietary antigen specific pTregs. Thus, it is likely that tools that deplete cells or delete genes using these markers also impact TCs. Interestingly, *Helicobacter*-specific Treg induction is superior in 3-week-old compared to 14-week-old mice [43], tracking with the developmental wave of TCs [170, 176]. However, despite the relative impairment of pTreg generation in older mice, even at 14 weeks old, a sizeable population of pTreg generations is produced [43], suggesting that either a distinct APC is responsible or that the smaller numbers of TCs are sufficient. Despite different approaches to identify tolDCs and TCs, these cells are highly similar and may represent overlapping cell types, and much work remains to clarify the relationship of these cells to cDCs. At present, such cells have yet to be purified in sufficient quantity to demonstrate this capacity in an *ex vivo* setting, likely due to the enormous technical challenges in doing so. Thus, while limiting MHCII expression to these cells appears to be sufficient for pTreg generation, it is not yet clear if this occurs in concert with other cell types for full maturation of the response. Although a role for AIRE + JCs in mediating pTreg development in response to the microbiome has not been established, the depletion of AIRE + APCs negates pTreg generation in response to oral antigen, although this does not require the activity of AIRE itself [171].

Despite many similarities between the induction of tolerance to orally-provided antigen and microbiome members, restricting MHCII expression to RORγt+ cells is sufficient to support robust *H. hepaticus*-specific RORγt+ pTreg development [167]. In contrast, RORγt+ cell expression of MHCII is only sufficient to induce FoxP3 but not RORγt expression in response to orally-provided antigen [178], and cDCs may additionally contribute to the full maturation of these cells. While mechanisms that mediate the tolerogenic effects of these cells are not fully defined, expression of components of the Transforming Growth Factor (TGF)-β activating αvβ8 integrin in CD11c+ cells (αv) [167] or RORγt+ cells (β8) [170] is critical for pTreg induction. Furthermore, using a mixed bone-marrow chimaera approach that allowed for the generation of mice where APCs could either present MHCII or activate TGF-β via αvβ8 integrin, but not both, it was shown that TGF-β activation and antigen presentation must be performed by the same cell type [170]. TGF-β has long been linked to the induction of Tregs from naïve precursors [179], however it has been challenging *in vivo* to distinguish between direct effects of TGF-β on Treg induction, from the pro-inflammatory environment induced following neutralization of the effects of TGF-β [180, 181]. The transfer of *Helicobacter*-specific CD4+ T cells expressing a dominant negative TGFβRII and thus having impaired responsiveness to TGF-β [180], into an otherwise normal host where all other cells received unimpaired levels of TGF-β stimulation, has suggested that the loss of TGF-β signaling in CD4+ T cells only has minor impacts on Treg differentiation [43]. The leakiness of the system used notwithstanding, these data suggest that rather than providing a signal for Treg development, the activation of TGF-β by TCs or PRDM16+ tolDCs might condition a tolerogenic phenotype in APCs in the face of stimulation with microbial products. This is evidenced by CD11c-Cre mediated deletion of the TGFβRI being linked with a reduced capacity to support pTreg development [182]. A prominent role has been shown for IL-10 in supporting development of microbiome-reactive Treg [44, 45], but it is not clear if this reflects a conditioning effect of IL-10 on the developing T cell, or the capacity of IL-10 to limit inflammatory responses in the face of microbial stimulation that would indirectly block Treg development. Notably, whether these novel APC possess the retinoic acid generating properties previously posited to drive Treg induction by intestinal DCs [150, 151] is unknown.

In the case of intestinal Th17 development, relatively little is known about the pathways inducing their development, however, for SFB-induced Th17 cells, IL-6 is required for early differentiation, but signals provided by other Signal Transducer and Activator of Transcription 3 (STAT3)-activating cytokines like IL-21 or IL-23 can support normative accumulation in the absence of IL-6 [161]. In addition, locally produced factors like serum amyloid A (SAA)1/2 are induced by SFB in epithelial cells and promote IL-17A expression in the lamina propria [137].

Despite these advances in our understanding of the APCs that induce tolerance, critical knowledge gaps still remain with respect to other cell fates. Although the role for TCs, and PRDM16+ tolDCs in priming *H. hepaticus*-specific pTreg is clear, *H. hepaticus*-specific FoxP3- T cells proliferate and acquire a Th17 cell phenotype in the absence of CCR7 or RORγt expression by CD11c+ or MHCII+ cells [167] or PRDM16-expression by RORγt+ cells [173]. This fact suggests that rather than a single APC type acquiring microbiome-derived antigen and polarizing the appropriate effector response based on features of the microbe, that instead, distinct APC types may acquire antigen and prime different responses to the same microbe. The identity of this APC that drives Th17 development in the absence of tolDC/TC is unknown, but both CD11c^low^CD70^high^ cells and migratory CD103-CD11b + DCs have previously been shown to induce Th17 cell responses and may represent a candidate APC type [152, 183]. Importantly, under homeostatic conditions, Th17 development among *H. hepaticus*-specific T cells is rare and is prevented by the CCR7+ APC-driven Treg development. However, whether CCR7+ APCs limit the acquisition of antigen by the Th17-priming subset under normal conditions, or whether their effect on CD4+ T cell fate is dominant, remains an open question. While the studies of SFB-driven Th17 cell development were largely performed prior to knowledge of TCs, tolDCs, or JCs, RORγt-driven deletion of MHCII enhances accumulation of Th17 cells in SFB-negative hosts but does not impact SFB-driven Th17 cell development [66]. Although originally thought to reflect the capacity of ILC3s to inhibit microbiome-reactive T cells [184], a failure to generate pTregs in these mice likely leads to enhanced differentiation and expansion of Th17 cells. Interestingly, while restriction of MHCII to CD11c+ cells supports robust differentiation of *H. hepaticus*-specific Tfh cells, restricting MHCII expression to RORγt+ cells does not. This suggests that the mixed CD4+ T cell fate that characterizes *H. hepaticus*-specific CD4+ T cells requires the function of distinct APCs. It is thus clear that there is unappreciated complexity that contributes to intestinal CD4+ T cell fate. Indeed, most work to date has focused on the cell type priming the initial response. However, it is possible that once activated, cells may modulate their output based on subsequent APCs they interact with, in addition to their location. SFB-specific cells acquire RORγt expression in MLNs, but maximal IL-17A secretion is triggered upon entry to the small intestine [137]. Similarly, *H. hepaticus-*specific CD4+ T cells adopt a Treg fate in the MLNs, but maximum effector function, including IL-10 expression, occurs in the lamina propria even when Treg development does not occur [166]. Thus, much work remains to be done to fully understand the complexity of APC antigen acquisition and presentation to CD4+ T cells in the context of microbiome-derived antigen.

## Polarizing factors

**Figure 2:**
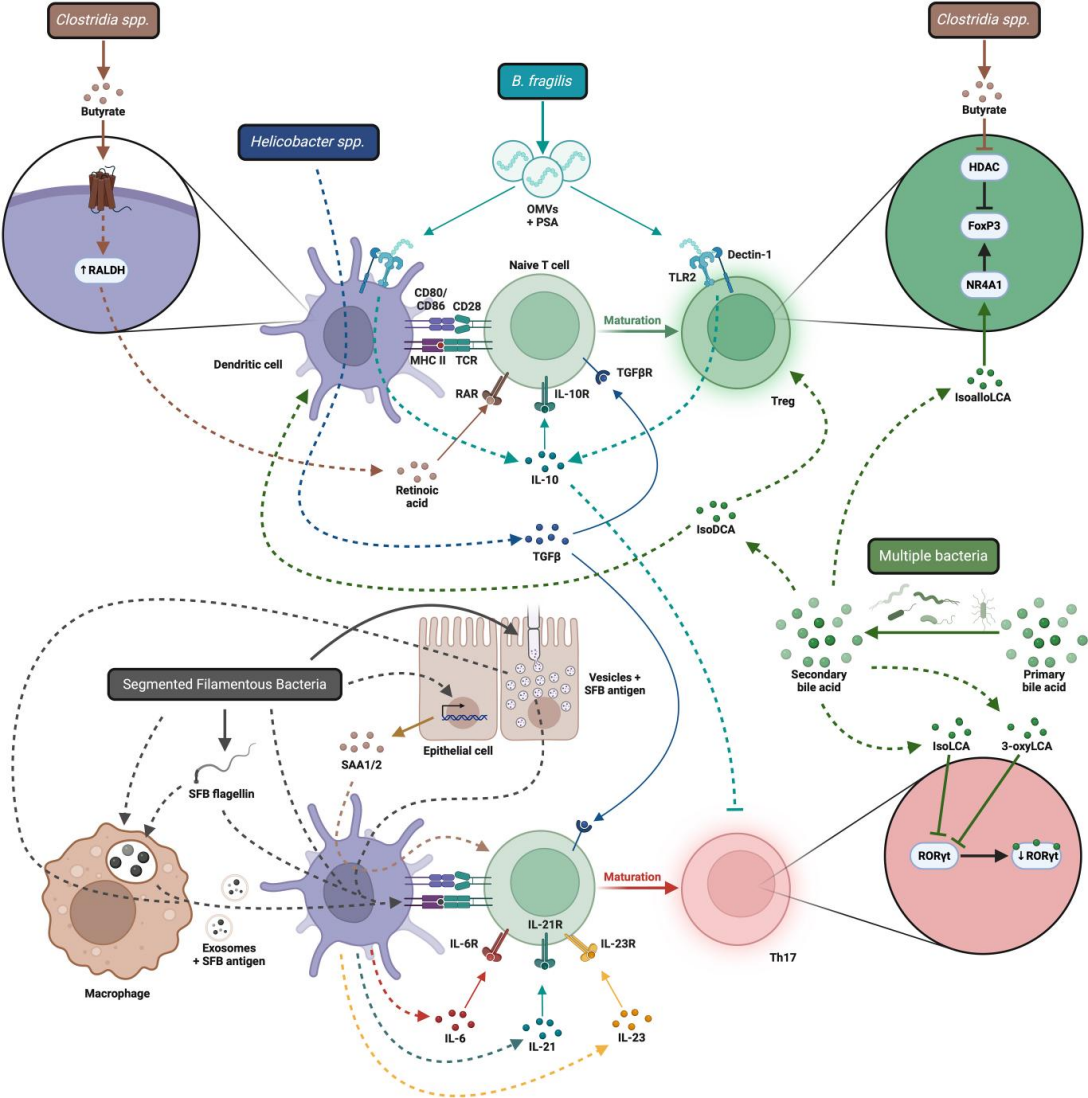
T cell and APC interactions leading to polarization of effector CD4+ T cells. Created in BioRender. Engelhart, M. (2025) https://BioRender.com/arug0to.

Concurrently with stimulation of the CD4+ T cell through its TCR via antigens presented on MHCII, APCs integrate information about the nature of the agent whose antigens they are presenting to coordinate the development of T cells with an appropriate effector phenotype (Fig. 2). This qualitative assessment is largely mediated through the expression of PRRs which sense conserved microbial patterns that convey broad phylogenetic information such that the APC can then provide signals, often secreted cytokines, to appropriately tailor the T cell response that ensues to adequately deal with the agent of interest [26, 185].

The prototypic immunomodulatory microbiome-derived product is polysaccharide A (PSA), a capsular zwitterionic polysaccharide that is produced by *B. fragilis* [186, 187]. PSA is packaged into OMVs [119] and can induce the development of IL-10-secreting Tregs that limit Th17 cell development [125, 188, 189] in response to *B. fragilis*. The recognition of PSA is mediated through the dual engagement of Dectin-1 and Toll-like receptor (TLR)2 which recognize the polysaccharide component and covalently attached lipid moiety of PSA, respectively [41, 188, 190–192]. Strikingly, PSA is sufficient to mediate many of the beneficial effects of *B. fragilis*, and its expression is required for optimal induction of Tregs by *B. fragilis* [41, 189, 191]. More recently, cell surface polysaccharides from *Bifidobacterium bifidum* [47] and *H. hepaticus* [193] have been linked to the induction of anti-inflammatory responses in a TLR2-dependent but Dectin-1-independent manner [47, 193], while the presence of a capsule in *Ruminococcus gnavus* strains has been linked to enhanced anti-inflammatory intestinal responses [194]. *B. bifidum* induces the development of IL-10+ pTregs in the colonic lamina propria, mediated through cell surface β-glucan and galactan polysaccharides which induce a TLR2-dependent tolerogenic phenotype in DCs [47]. While the specific IL-10-inducing factor produced by *H. hepaticus* has not yet been identified, biochemical assays suggest it is carbohydrate in nature, and is also sensed through TLR2 [193]. Expression of IL-10 is critical to limit the development of pathogenic CD4+ T cell responses to *H. hepaticus*, suggesting that such carbohydrate factors are required to induce an anti-inflammatory response. While information regarding PRR agonists from other microbiome members that coordinate CD4+ T cell function is scant, the induction of colonic Tregs by ASF and IL-10-secreting Tr1 cells by *Bifidobacterium breve* rely on recognition by host-encoded PRR, being MyD88- and Ticam-dependent, and TLR2- and MyD88-dependent [31, 48], respectively. Outside of TLR2-mediated recognition, TLR9-mediated sensing of microbiome-derived DNA can inhibit Treg accumulation in the colon, and strikingly, select microbiome members such as *Lactobacillus spp.* can inhibit TLR9 recognition to facilitate Treg generation. By appropriately regulating the size of the Treg compartment through TLR9, distinct microbes can maintain resistance to pathogens by tuning the ratio of Tregs to effector T cells [195], while others can block TLR9 stimulation to sustain Tregs which is protective in the context of intestinal infection [196]. Much less is known about the nature of the microbial signals that promote Th17 differentiation, but intraperitoneal administration of SFB flagellin has been shown to induce Th17-related responses in intestinal tissues [197].

The paucity of cell wall products that have been biochemically characterized has limited our understanding as to whether intestinal homeostasis is mediated by an assortment of microbiome-derived TLR2 agonists or by a single universal agonist common to many microbes. Furthermore, although stimulation of TLR2 via microbiome-derived agonists plays a prominent role in inducing immune homeostasis through their impact on T cells, TLR2 also mediates resistance to intestinal pathogens [198], and thus sensing of TLR2 agonists alone is insufficient to explain the connection between TLR2 and immune tolerance. Thus, biochemical characterization of a greater number of microbiome-derived agonists is needed. However, the challenges associated with biochemical purification of individual components from whole cells have long confounded the identification of PRR agonists [199–201]. In the case of PSA, the TLR2-stimulatory capacity was long-attributed to the polysaccharide component until a previously unappreciated covalently attached lipid was identified as key to the IL-10 inducing capacity of PSA [190]. Coupled with the propensity of TLR2 to sense lipids, this suggests that perhaps the activity of additional cellular products that operate via TLR2 are dependent on the presence of lipids that may have been overlooked. Efforts to study the necessity of these factors through gene deletion have been limited without the genetic characterization and technical understanding to delete the genes that coordinate their production, many of which likely cannot be deleted as they encode products that are sensed specifically because they cannot be turned off which would allow for easy immune evasion. Although it has been posited that such molecules may represent inherently tolerance-inducing entities, PSA was originally described as a driver of abdominal abscesses [187], and thus it is likely that the nature of the encounter, not simply the biochemical features of such molecules, dictates the development of tolerance. Much work remains to be done to better understand how these factors shape immune responses to microbiome members and to determine if there is a common biochemical feature that truly favors, or is conducive to, induction of CD4+ T cell tolerance.

The second class of molecules that guide the coordination of helper T cell function upon activation are metabolites that are produced or modified by the microbiome (referred to simply as microbial/microbiome metabolites hereafter). Short-chain fatty acids (SCFAs) are among the most prominent mediators of microbiome impacts on the host. SCFAs are the product of microbial fermentation of fiber, resistant starches, and otherwise indigestible carbohydrates, and represent one of the canonical functions provided by the gut microbiome [202, 203]. Intestinal SCFAs are dominated by acetate, propionate and butyrate, and are almost entirely absent in germ-free mice [202]. SCFAs reach their highest concentrations in the colon and although they have now been linked to phenotypic imprinting of numerous cell types [203], one of their earliest ascribed roles in immunomodulation was that of Treg-induction [204–206]. While the impact of genetic ablation of SCFA-producing ability on immune function has not been reported to our knowledge, the exogenous administration of butyrate, propionate or acetate can all promote Treg accumulation in the colon or the MLN [204–206], although the precise site affected differs between studies. The specific SCFAs produced varies across different classes of bacteria with acetate produced by a wide range of microbes, while propionate and butyrate production are more specialized. This redundancy with respect to Treg generation likely ensures that Treg-inducing capacity can be maintained across distinct microbial community structures where the precise composition of the SCFA pool may vary [207, 208]. Mechanistically, SCFAs mediate their functions both through sensing by specific host-encoded receptors as well as through modification of cellular epigenetic state and epigenetic regulators. Propionate and butyrate both engage host-expressed receptors to drive Treg accumulation in the colon, with propionate acting through GPR43 [204] and butyrate through GPR109a [209] (also a receptor for niacin). However, following passive diffusion or Slc5a8-mediated uptake [210], both propionate and butyrate can also directly inhibit the function of histone deacetylases (HDAC) through chemical modification [211, 212]. Furthermore, butyrate can provide a source of acetyl-CoA that leads to enhanced histone acetylation [213]. These mechanisms have been implicated in the capacity of butyrate to enhance histone H3 acetylation at the FoxP3 promoter [206] and conserved noncoding sequence (CNS) 1 and CNS3 [205, 206], which support the peripheral induction and enhance the efficiency of FoxP3 expression respectively [214]. Although butyrate can directly promote FoxP3 expression in naïve T cells [205, 206], it can also modify DC function to promote Tregs through inhibition of HDACs [205]. Despite a variety of studies replicating these findings, other studies have found no role for SCFAs in mediating Treg induction [33]. Furthermore, how the effects of SCFAs are limited to induction of FoxP3 and not to other CD4+ T cell fate-specifying transcription factors [206] remains unclear, but suggest that additional unknown factors are involved.

Another class of molecules which affect host physiology and are modified by the gut microbiome are bile acids. Bile acids are a set of compounds generated by the host from cholesterol in the liver that are secreted into the intestine to aid in lipid absorption. These primary bile acids can be modified by members of the microbiome to generate secondary bile acids, which can in turn shape host physiology through recognition by host-encoded receptors. Most bile transformations are mediated by the actions of bile salt hydrolases (BSH), which are widely distributed among members of the microbiome, and 7-α-dehydroxylases, which are less prevalent [215, 216]. The effects of the microbiome on bile varies highly depending on the microbe performing the transformation. Furthermore, due to the recycling of secondary bile acids, multiple distinct microbes may modify a given bile molecule, and thus the pool of bile acids varies across individuals in a microbiome-dependent manner [215]. Given the broad impact of secondary bile acids on host physiology, their capacity to shape immune function has received considerable attention, although only a handful of microbially-modified bile acids have been shown to shape intestinal T cell responses. The exogenous administration of derivatives of lithocholic acid (LCA) is sufficient to both inhibit Th17 development and promote Treg accumulation. 3-oxoLCA and isoLCA, which are generated by the action of 3α- and 3β- hydroxysteroid dehydrogenase enzymes, antagonize the Th17-specifying transcription factor RORγt in CD4+ T cells to limit Th17 cell development [217, 218]. Another LCA derivative, isoalloLCA, actively promotes the expression of FoxP3 through the T cell-expressed nuclear receptor NR4A1 in a CNS3-dependent, but CNS1-independent, manner [217, 219], likely through enhancing the acetylation of the FoxP3 locus. Notably, the capacity of *B. fragilis* and *Bacteroides thetaiotaomicron* to modify bile acid pools via BSH but not 7-α-hydroxysteroid dehydrogenase, has been critically linked to the accumulation of colonic RORγt+ pTregs [220]. The generation of isoDCA by co-metabolism of bile by 7α-hydroxyl cleavage from cholic acid by *Clostridium scindens* and the 3α-hydroxyl epimerization activity of *Ruminococcus gnavus* hydroxysteroid dehydrogenases heterologously expressed in *B. thetaiotaomicron* also drives RORγt+ Treg accumulation [221]. As with SCFAs however, data showing a requirement rather than sufficiency for these molecules is currently lacking due to significant challenges associated with the genetic modification of many members of the microbiome. Collectively, these studies reinforce the notion that many secondary bile acids are indeed critical regulators of the CD4+ T cell phenotype in the intestine.

Other metabolites have also been linked to polarization of intestinal T cells fate, albeit much less is known about these molecules. Microbiome-derived adenosine triose phosphate (ATP) can stimulate CD11c^_low_^ CD70^high^ APCs to induce the accumulation of Th17 cells [183], although it is unclear given the ubiquity of ATP production if all microbes are capable of supporting Th17 cell development via ATP generation. Tryptophan availability has long been appreciated to represent a critical regulator of immunity. While its effects can be traced in part to sensing of its availability by the GCN2 kinase [222], the metabolism of tryptophan to generate products which agonize the aryl hydrocarbon receptor (AhR), also plays important roles in its effects on the immune system [223]. AhR is expressed in intestinal pTregs and controls accumulation of intestinal Tregs, suggesting that a microbiome-derived metabolite contributes to intestinal Tregs through AhR [224]. *Lactobacillus reuteri-*produced indole-3-lactic acid (ILA) induces the accumulation of CD4+CD8αα+ IEL in an AhR-dependent manner [225], while indole-3-propionic acid promotes mitochondrial respiration in intestinal CD4+ T cells in an AhR-independent manner, limiting their capacity to differentiate into Th1 or Th17 cells [226]. Finally, gut microbes have recently been shown to produce serotonin which can promote Treg generation [227]. Thus, the microbiome generates a broad array of secreted molecules that can shape CD4+ T cell phenotypes to promote immune tolerance.

The recognition of cell wall components is understood to allow direct linking of the antigen source to the specific PRR agonist. As these molecules are often physically linked and thus enter the same phagosome, this often links a particular effector response to select antigens [228]. Metabolites, on the other hand, represent secreted/released effectors, and therefore, their impact is likely not directly linked to T cells specific for the microbes producing the metabolites. Indeed, butyrate has been directly shown to support the development of Tregs to orally administered antigen [206]. Thus, metabolites likely broadly condition the intestinal environment to favour T cell responses that maintain tissue integrity and mutualistic interactions. As such, the sensing of these metabolites may act akin to effector-triggered immunity [229], whereby the presence of these metabolites indicates a healthy microbiome state to which the host immune system should be tolerant without direct sensing of all the community members. Furthermore, this would ensure tolerance toward myriad distinct microbiome compositions, so long as they provided benefits to the host in the absence of danger [230], and lack of these molecules may indicate pathogenic disruptions that necessitate a distinct CD4+ T cell response. Interestingly, although a Th1-promoting environment can drive microbiome-reactive CD4+ T cells toward a Th1 phenotype [231], SFB-reactive T cells maintain a Th17 bias when responding to SFB even in the face of a concurrent strongly pro-Th1 response [232]. Thus, the Th17-inducing signals of SFB are likely delivered together with its antigens and can either overcome pro-Th1 signals or occur in an anatomic location where such signals are not present. Understanding how these two distinct scenarios are controlled will be important for the delivery of tolerance-promoting therapies that by necessity will most often be administered in the context of ongoing inflammatory responses.

## Antigen specificity

Irrespective of the specific APC presenting microbiome-derived antigen or the signals conditioning the outcome of the response, antigen-bearing APCs must ultimately encounter CD4+ T cells with a TCR specific for these antigens to activate and polarize a CD4+ T cell response. Deciphering whether the CD4+ T cell subsets that accumulate in response to the microbiome are specific for the inducing agent or whether they are comprised of cells with a range of specificities, including the inducing microbe and other antigens in the environment, is of fundamental importance. Two distinct, but not mutually exclusive, possibilities exist for microbiome-modulated CD4+ T cell differentiation. First, specific microbes may generate an environment that promotes particular effector types, even if the antigens are derived from microbes distinct from those conditioning the environment. The second possibility is that T cells of a particular effector class are specific only for the microbe that provides the polarizing signals. While the latter model has been thought to explain how the immune system avoids mounting inappropriate responses to distinct microbial entities, as discussed below, examples of environmental conditioning have been uncovered and are in keeping with the role of microbially produced or modified molecules like metabolites in driving CD4+ T cell fate.

Seminal studies on the emergence of microbiome-specific T cells came from interrogation of the ability of the pathobiont *H. hepaticus* to shape CD4+ T cell function. Using a combination of approaches, it was established that *H. hepaticus* elicited the development of *H. hepaticus*-specific Tregs with anti-colitic activity in the MLN [44]. Moreover, when key regulatory pathways were disabled in the context of a dysfunctional IL-10 axis, colitogenic *H. hepaticus*-specific CD4+ T cell populations expressing IL-17A and IFN-γ emerged that could drive intestinal pathology [233–236]. More recently, the development of TCR transgenic mice specific for gut microbiome encoded-antigens has reinforced these findings, revealing that CD4+ T cells with specificity for SFB [232], *B. thetaiotaomicron* [42], *Helicobacter spp.* (*H. apodemus*, *H. hepaticus*, and *H. typhlonius*) [43, 45, 46], and *A. muciniphila* [72] leads to the development of Th17 cells, Tregs, and/or Tfh in the intestine under homeostatic conditions. Moreover, *ex vivo* assays have further shown that a cocktail of *Clostridia spp.* [13] and *B. bifidum* [47] also induce the accumulation of *Clostridia*-specific or *B. bifidum*-specific Tregs, respectively. Collectively, these studies have provided unequivocal evidence that gut microbes can drive the differentiation of CD4+ T cells that are specific for antigens that they encode. However, what has remained less clear is whether these microbes create a milieu that favors the development of specific CD4+ T cell lineages in response to antigen exposure irrespective of the source of antigen, or if these microbes coordinate development of such cells specific solely for their own antigens.

While challenging to address, a limited number of studies support the notion that these microbes may promote an environment conducive to the polarization of desired effector classes. In the context of SFB-induced Th17 differentiation, *ex vivo* assessment of the specificity of Th17 cells using hybridoma based systems revealed that, although SFB-specific cells are highly abundant within the pool of intestinal Th17 cells, a significant number of Th17 cells specific for non-SFB antigens also develop [66, 232]. Although it is possible that these Th17 cells represented those that had differentiated prior to SFB colonization rather than being elicited by colonization with SFB, their preponderance in the Th17 pool (>50% of the tested fusions were not SFB-reactive in one study [66]) was disproportionate with the Th17 cell abundance in mice lacking SFB (Th17 abundance increased >5-fold following SFB colonization). The adoptive transfer of T cells specific for a non-SFB antigen has provided a more formal test of whether SFB induces these cells, but has also failed to provide a clear answer. In one such study, the adoptive transfer of CD4+ T cells from ovalbumin-reactive OT-II and melanocyte-reactive TRP-1 TCR transgenic mouse lines, Th17 development in the transgenic T cells was unaffected by SFB colonization, even with provision of their cognate antigen [66]. Conversely, using a similar experimental framework with OT-II TCR transgenic CD4+ T cells, a separate study found that SFB could enhance Th17 differentiation among transferred OT-II cells when ovalbumin was provided [65]. While this latter study did not use recombination activating gene (*Rag*)*-*/*-* OT-II, and thus could not completely exclude a role for additional specificities in the transferred cells due to secondary TCR rearrangement, stimulation with ovalbumin was required for Th17 development suggesting that triggering of a non-SFB TCR was critical for the response. Similarly, provision of the SCFA butyrate, promotes the development of ovalbumin-stimulated OT-II into Tregs in the colon [206], suggesting some microbiome members elicit an environment that is conducive to Treg development rather than simply inducing Treg development to their own antigens exclusively.

An additional layer of complexity in the study of antigen specificity is the inherent cross-reactivity of the TCR [237]. While the antigen-specificity of intestinal T cells has been largely studied in the context of individual microbes, it is likely that individual T cell clones harbor reactivity to other microbiome members due to shared epitopes and cross-reactivity. Although not extensively explored, several studies support a model whereby intestinal CD4+ T cell clones are reactive to multiple different species. This was first established for *H. hepaticus* using CD4+ T cell clones generated from *H. hepaticus*-colonized mice that are specific for the flagellar hook protein which were shown to be reactive to some, but not all, closely related *Helicobacter* species [238. A similar phenomenon was uncovered using a *B. thetaiotaomicron*-specific hybridoma generated from CD4+ T cells that are specific for an epitope derived from BT0900, but were also reactive to other *Bacteroides* species that had exact copies of this epitope [239]. More recently, a systematic study of the antigen specificity of intestinal CD4+ T cells expanded on these findings by identifying epitopes from a single antigen that were found in multiple distinct microbiome members and recognized by highly represented TCRs within the pool of intestinal CD4+ T cells [240]. Collectively, these data suggest that individual T cell clones can harbor specificity for several microbiome members, negating the need for a dedicated pool of cells with reactivity to individual strains. This points to a mechanism through which intestinal CD4+ T cells maintain broad reactivity to the enormous microbial complexity of the gut microbiome within the constraints of the available TCR repertoire and limitations on the size of the CD4+ T cell compartment.

Despite this evidence, it is unclear how the intestinal immune system could tailor the quality of responses appropriate for different microbiome members if there is wide-spread expression of the epitope for which the T cell is specific. In such a scenario, a Treg response targeted to one microbe may not be appropriate for a different microbe that shares the same epitope. If epitope sharing is most common among closely related species, the likelihood that deleterious agents gain the benefit of epitope sharing with beneficial microbes is limited. However, even closely related strains of *B. fragilis* can exert distinct impacts [41, 241] on the host depending on the presence of the *B. fragilis* toxin (BFT), suggesting that tuning the response at the level of individual isolates may be useful. Second, the mere presence of the DNA sequence that encodes the epitope in the genome does not guarantee recognition as *B. thetaiotaomicron*-specific T cells which recognize the antigen encoded by *BT0900*, fail to respond to *B. caccae* despite this bacterium sharing the identical epitope [239]. Similar findings have been reported for BθOM mice that express a *B. thetaiotaomicron*-specific MHC II-restricted TCR that responds to a *BT4295*-encoded epitope. In this model system it has been demonstrated that many, but not all, strains of *B. thetaiotaomicron* with the cognate epitope are recognized by the TCR [242].

To further complicate matters of antigen specificity, TCRs may also recognize both microbiome-encoded and self-antigens. Striking data have implicated a role for SFB as a driver of autoimmune pathology in extra-intestinal sites, including the pancreas [243], joints [244], and central nervous system [245, 246]. Although this could be mediated independently of SFB-specific Th17 cells, a recent study identified SFB-reactive Th17 cells that recognize self-antigens expressed in the central nervous system (the receptor tyrosine kinase ERBB2, trophinin 1, ANAPC2) that can induce central nervous system pathology [246]. A similar phenomenon has been demonstrated in a murine model of uveitis caused by CD4+ T cell reactivity to interphotoreceptor retinoid-binding protein (IRBP). IRBP-specific Th17 cells developed in the intestine in a microbiome-dependent, but IRBP-independent, manner, implicating cross-reactivity between IRBP and a microbiome-encoded antigen [247]. Notably, this was not thought to require SFB colonization. Little is known about the cross-reactivity between microbiome-reactive Treg TCRs and self-antigens, but it has been suggested that T cells with microbiome-reactive TCRs can acquire FoxP3 expression in the thymus [248]. This is in keeping with a study that identified tTreg development driven by a TCR with non-self-cognate specificity [249]. Whether the self-reactivity of microbiome-reactive TCR plays a role in their thymic selection or peripheral maintenance is unclear. However, at least in the case of SFB-reactive T cells, the affinity of the TCR for self-antigen is lower than for SFB-encoded antigens, and their pathogenic functions with respect to autoimmunity likely require priming by a microbial agent to generate the conditions to support their pathogenic function.

## Conclusions

While study of the microbiome-driven differentiation of CD4+ T cell fate in the intestine has provided myriad insights into how homeostatic responses against non-genomically encoded antigens can be achieved, many questions remain to be answered to fully comprehend how the delicate balance of CD4+ T cell tolerance and effector functions is maintained. Our limited capacity to genetically modify microbiome members has contributed to our incomplete understanding of how microbiome members shape intestinal immune responses. Furthermore, despite the wealth of information that has been gathered to date, current knowledge is built off the study of a small pool of model organisms. While many of these findings may be broadly applicable to other bacterial species, a critical challenge of future work will lie in validating how universal these mechanisms of antigen acquisition, presentation, and induction of effector function are across the repertoire of microbes in our gut, and indeed, whether these mechanisms extend beyond the recognition of bacterial members of the microbiome. The renewed interest in oral tolerance [21] has revealed several overlapping features with induction of tolerance to the microbiome. Despite many similarities, differences in the requirements for tolerance to oral or microbial antigens exist [167, 178], and illumination of these differences will further advance our understanding of intestinal immune tolerance. Given the vast complexity of the microbiome, the task of unraveling these questions is undoubtably immense, but is of paramount importance in the continued effort to understand the relationship between the intestinal immune system and the microbiome. Moreover, as intestinal pTregs can limit responses to oral vaccines, the capacity to limit their development in select time windows may be of benefit [250]. The studies discussed here focused on work from murine models, which have provided the tools necessary to derive insights into the nature of CD4+ T cell-microbiome interactions. Emerging evidence suggests that these relationships are not unique to mice. Microbiome-specific CD4+ T cells are prevalent in healthy humans [251], and human-derived gut microbes coordinate the development of Treg [13, 32] and Th17 responses [13, 34] in mice, suggesting conservation of these responses between the two species. However, more studies are required to delineate the impact of specific gut microbiome members on intestinal T cell responses in humans. Considering the emerging evidence that the microbiome plays a pivotal role in myriad inflammatory diseases, both inside and outside of the intestine, understanding the relationship between intestinal T cells and the microbiome is critical to the development of durable therapeutic strategies that tailor interactions between host and microbe to promote/restore immune tolerance.

## Data Availability

Not applicable.

## Permission to reproduce

Figures were prepared using BioRender, license Engelhart, M. (2025) https://BioRender.com/s6vv57i and https://BioRender.com/arug0to

## Abbreviations

AhR: aryl hydrocarbon receptor
AIRE: autoimmune regulator
APC: antigen presenting cell
ASF: altered Schaedler Flora
ATP: adenosine triose phosphate
BFT: *B. fragilis* toxin
BSH: bile salt hydrolase
CCR2: C-C chemokine receptor 2
CCR7: C-C chemokine receptor 7
cDC: conventional/classical dendritic cell
CP: cryptopatch
CNS: conserved noncoding sequence
CSF: colony stimulating factor
DC: dendritic cell
DT: diphtheria toxin
DTR: diphtheria toxin receptor
EGFR: epidermal growth factor receptor
FAE: follicular associated epithelium
Flt3l: FMS-like tyrosine kinase 3 ligand
GALT: gut-associated lymphoid tissues
GAP: goblet cell-associated antigen passages
HDAC: histone deacetylase
IEC: intestinal epithelial cell
IgA: immunoglobulin A
IL-: interleukin
ILA: indole-3-lactic acid
ILC3: type 3 innate lymphoid cell
ILF: isolated lymphoid follicle
IRBP: interphotoreceptor retinoid-binding protein
JC: Janus cell
LCA: lithocholic acid
LTα: lymphotoxin alpha
M Cell: microfold cell
MHC: major histocompatibility complex
MLN: mesenteric lymph nodes
MV: membrane vesicle
OMV: outer membrane vesicle
PP: Peyer’s patches
PRR: pattern recognition receptor
PSA: polysaccharide A
pTreg: peripheral regulatory T cell
RAG: recombination activating gene
RORα: retinoic acid receptor–related orphan receptor alpha
RORγt: retinoic acid receptor-related orphan receptor gamma t
SAA: serum amyloid A
SCFAs: short chain fatty acids
SFB: segmented filamentous bacteria
SILT: solitary intestinal lymphoid tissue
STAT: signal transducer and activator of transcription
TC: Thetis Cells
TCR: T cell receptor
TED: trans epithelial dendrite
Tfh: T follicular helper cell
TGF-β: transforming growth factor
Th: T helper cell
TLR: toll-like receptor
tolDC: tolerogenic dendritic cell
Tr1: T regulatory 1 cell
Treg: regulatory T cell
tTreg: thymic regulatory T cell

## References

[1] Owen RD . Immunogenetic consequences of vascular anastomoses between bovine twins. Science 1945, 102, 400–1. 17755278 10.1126/science.102.2651.400

[2] Billingham RE, Lampkin GH, Medawar PB, Williams HL. Tolerance to homografts, twin diagnosis, and the freemartin condition in cattle. Heredity (Edinb) 1952, 6, 201–12.

[3] Burnet FM . The Production of Antibodies. New York: Macmillan, 1941.

[4] Burnet FM, Fenner F. The Production of Antibodies (2nd edn). In: Burnet FM, Fenner F (eds). Melbourne: Macmillan, 1949.

[5] Billingham RE, Brent L, Medawar PB. Actively acquired tolerance of foreign cells. Nature 1953, 172, 603–6. 13099277 10.1038/172603a0

[6] Janeway CA Jr . Frontiers of the immune system. Nature 1988, 333, 804–6. 2968519 10.1038/333804a0

[7] Blumberg RS, MacDonald TT. Mucosal immunology: a frontier no longer. Mucosal Immunol 2008, 1, 3.

[8] Mowat AM . To respond or not to respond—a personal perspective of intestinal tolerance. Nat Rev Immunol 2018, 18, 405–15. 29491358 10.1038/s41577-018-0002-x

[9] Bäckhed F, Ley RE, Sonnenburg JL, Peterson DA, Gordon JI. Host-bacterial mutualism in the human intestine. Science 2005, 307, 1915–20. 15790844 10.1126/science.1104816

[10] Ley RE, Lozupone CA, Hamady M, Knight R, Gordon JI. Worlds within worlds: evolution of the vertebrate gut microbiota. Nat Rev Microbiol 2008, 6, 776–88. 18794915 10.1038/nrmicro1978 PMC2664199

[11] Ley RE, Peterson DA, Gordon JI. Ecological and evolutionary forces shaping microbial diversity in the human intestine. Cell 2006, 124, 837–48. 16497592 10.1016/j.cell.2006.02.017

[12] Relman DA . The human microbiome: ecosystem resilience and health. Nutr Rev 2012, 70 Suppl 1, S2–9. 22861804 10.1111/j.1753-4887.2012.00489.x PMC3422777

[13] Atarashi K, Tanoue T, Oshima K, Suda W, Nagano Y, Nishikawa H, et al Treg induction by a rationally selected mixture of Clostridia strains from the human microbiota. Nature 2013, 500, 232–6. 23842501 10.1038/nature12331

[14] Atarashi K, Tanoue T, Shima T, Imaoka A, Kuwahara T, Momose Y, et al Induction of colonic regulatory T cells by indigenous Clostridium species. Science 2011, 331, 337–41. 21205640 10.1126/science.1198469 PMC3969237

[15] Gaboriau-Routhiau V, Rakotobe S, Lecuyer E, Mulder I, Lan A, Bridonneau C, et al The key role of segmented filamentous bacteria in the coordinated maturation of gut helper T cell responses. Immunity 2009, 31, 677–89. 19833089 10.1016/j.immuni.2009.08.020

[16] Ivanov II, Atarashi K, Manel N, Brodie EL, Shima T, Karaoz U, et al Induction of intestinal Th17 cells by segmented filamentous bacteria. Cell 2009, 139, 485–98. 19836068 10.1016/j.cell.2009.09.033 PMC2796826

[17] Ivanov II, Frutos Rde L, Manel N, Yoshinaga K, Rifkin DB, Sartor RB, et al Specific microbiota direct the differentiation of IL-17-producing T-helper cells in the mucosa of the small intestine. Cell Host Microbe 2008, 4, 337–49. 18854238 10.1016/j.chom.2008.09.009 PMC2597589

[18] Talham GL, Jiang HQ, Bos NA, Cebra JJ. Segmented filamentous bacteria are potent stimuli of a physiologically normal state of the murine gut mucosal immune system. Infect Immun 1999, 67, 1992–2000. 10085047 10.1128/iai.67.4.1992-2000.1999 PMC96557

[19] Mowat AM, Agace WW. Regional specialization within the intestinal immune system. Nat Rev Immunol 2014, 14, 667–85. 25234148 10.1038/nri3738

[20] Mowat AM . Anatomical basis of tolerance and immunity to intestinal antigens. Nat Rev Immunol 2003, 3, 331–41. 12669023 10.1038/nri1057

[21] Cerovic V, Pabst O, Mowat AM. The renaissance of oral tolerance: merging tradition and new insights. Nat Rev Immunol 2025, 25, 42–56. 39242920 10.1038/s41577-024-01077-7

[22] Ansaldo E, Farley TK, Belkaid Y. Control of immunity by the microbiota. Annu Rev Immunol 2021, 39, 449–79. 33902310 10.1146/annurev-immunol-093019-112348

[23] Macpherson AJ, Yilmaz B, Limenitakis JP, Ganal-Vonarburg SC. Iga function in relation to the intestinal microbiota. Annu Rev Immunol 2018, 36, 359–81. 29400985 10.1146/annurev-immunol-042617-053238

[24] Ost KS, Round JL. Communication between the microbiota and mammalian immunity. Annu Rev Microbiol 2018, 72, 399–422. 29927706 10.1146/annurev-micro-090817-062307 PMC7294967

[25] Torow N, Hand TW, Hornef MW. Programmed and environmental determinants driving neonatal mucosal immune development. Immunity 2023, 56, 485–99. 36921575 10.1016/j.immuni.2023.02.013 PMC10079302

[26] Ruterbusch M, Pruner KB, Shehata L, Pepper M. In vivo CD4(+) T cell differentiation and function: revisiting the Th1/Th2 paradigm. Annu Rev Immunol 2020, 38, 705–25. 32340571 10.1146/annurev-immunol-103019-085803

[27] Bucy RP, Panoskaltsis-Mortari A, Huang GQ, Li J, Karr L, Ross M, et al Heterogeneity of single cell cytokine gene expression in clonal T cell populations. J Exp Med 1994, 180, 1251–62. 7523568 10.1084/jem.180.4.1251 PMC2191707

[28] Bucy RP, Karr L, Huang GQ, Li J, Carter D, Honjo K, et al Single cell analysis of cytokine gene coexpression during CD4+ T-cell phenotype development. Proc Natl Acad Sci U S A 1995, 92, 7565–9. 7638231 10.1073/pnas.92.16.7565 PMC41380

[29] Kiner E, Willie E, Vijaykumar B, Chowdhary K, Schmutz H, Chandler J, et al Gut CD4(+) T cell phenotypes are a continuum molded by microbes, not by T(H) archetypes. Nat Immunol 2021, 22, 216–28. 33462454 10.1038/s41590-020-00836-7 PMC7839314

[30] Honda K, Littman DR. The microbiota in adaptive immune homeostasis and disease. Nature 2016, 535, 75–84. 27383982 10.1038/nature18848

[31] Geuking MB, Cahenzli J, Lawson MA, Ng DC, Slack E, Hapfelmeier S, et al Intestinal bacterial colonization induces mutualistic regulatory T cell responses. Immunity 2011, 34, 794–806. 21596591 10.1016/j.immuni.2011.03.021

[32] Faith JJ, Ahern PP, Ridaura VK, Cheng J, Gordon JI. Identifying gut microbe-host phenotype relationships using combinatorial communities in gnotobiotic mice. Sci Transl Med 2014, 6, 220ra11. 10.1126/scitranslmed.3008051 PMC3973144 24452263

[33] Sefik E, Geva-Zatorsky N, Oh S, Konnikova L, Zemmour D, McGuire AM, et al MUCOSAL IMMUNOLOGY. Individual intestinal symbionts induce a distinct population of RORγ+ regulatory T cells. Science 2015, 349, 993–7. 26272906 10.1126/science.aaa9420 PMC4700932

[34] Tan TG, Sefik E, Geva-Zatorsky N, Kua L, Naskar D, Teng F, et al Identifying species of symbiont bacteria from the human gut that, alone, can induce intestinal Th17 cells in mice. Proc Natl Acad Sci U S A 2016, 113, E8141–E50. 27911839 10.1073/pnas.1617460113 PMC5167147

[35] Izcue A, Coombes JL, Powrie F. Regulatory lymphocytes and intestinal inflammation. Annu Rev Immunol 2009, 27, 313–38. 19302043 10.1146/annurev.immunol.021908.132657

[36] Brockmann L, Tran A, Huang Y, Edwards M, Ronda C, Wang HH, et al Intestinal microbiota-specific Th17 cells possess regulatory properties and suppress effector T cells via c-MAF and IL-10. Immunity 2023, 56, 2719–35.e7. 38039966 10.1016/j.immuni.2023.11.003 PMC10964950

[37] Kumar P, Monin L, Castillo P, Elsegeiny W, Horne W, Eddens T, et al Intestinal interleukin-17 receptor signaling mediates reciprocal control of the gut microbiota and autoimmune inflammation. Immunity 2016, 44, 659–71. 26982366 10.1016/j.immuni.2016.02.007 PMC4794750

[38] Majumder S, McGeachy MJ. IL-17 in the pathogenesis of disease: good intentions gone awry. Annu Rev Immunol 2021, 39, 537–56. 33577346 10.1146/annurev-immunol-101819-092536 PMC8603601

[39] Mills KHG . IL-17 and IL-17-producing cells in protection versus pathology. Nat Rev Immunol 2023, 23, 38–54. 35790881 10.1038/s41577-022-00746-9 PMC9255545

[40] Medawar P . 1960. Immunological Tolerance. Peter Medawar—Nobel Lecture. NobelPrize.org. Nobel Prize Outreach 2025. https://www.nobelprize.org/prizes/medicine/1960/medawar/lecture/ (24 October 2025, date last accessed).

[41] Round JL, Mazmanian SK. Inducible Foxp3+ regulatory T-cell development by a commensal bacterium of the intestinal microbiota. Proc Natl Acad Sci U S A 2010, 107, 12204–9. 20566854 10.1073/pnas.0909122107 PMC2901479

[42] Wegorzewska MM, Glowacki RWP, Hsieh SA, Donermeyer DL, Hickey CA, Horvath SC, et al Diet modulates colonic T cell responses by regulating the expression of a *Bacteroides thetaiotaomicron* antigen. Sci Immunol 2019, 4, eaau9079. 30737355 10.1126/sciimmunol.aau9079 PMC6550999

[43] Nutsch K, Chai JN, Ai TL, Russler-Germain E, Feehley T, Nagler CR, et al Rapid and efficient generation of regulatory T cells to commensal antigens in the periphery. Cell Rep 2016, 17, 206–20. 27681432 10.1016/j.celrep.2016.08.092 PMC5051580

[44] Kullberg MC, Jankovic D, Gorelick PL, Letterio CP, Cheever JJ, W A, et al Bacteria-triggered CD4(+) T regulatory cells suppress Helicobacter hepaticus-induced colitis. J Exp Med 2002, 196, 505–15. 12186842 10.1084/jem.20020556 PMC2196050

[45] Xu M, Pokrovskii M, Ding Y, Yi R, Au C, Harrison OJ, et al c-MAF-dependent regulatory T cells mediate immunological tolerance to a gut pathobiont. Nature 2018, 554, 373–7. 29414937 10.1038/nature25500 PMC5814346

[46] Chai JN, Peng Y, Rengarajan S, Solomon BD, Ai TL, Shen Z, et al *Helicobacter* species are potent drivers of colonic T cell responses in homeostasis and inflammation. Sci Immunol 2017, 2, eaal5068. 10.1126/sciimmunol.aal5068 PMC5684094 28733471

[47] Verma R, Lee C, Jeun EJ, Yi J, Kim KS, Ghosh A, et al Cell surface polysaccharides of *Bifidobacterium bifidum* induce the generation of Foxp3(+) regulatory T cells. Sci Immunol 2018, 3, eaat6975. 10.1126/sciimmunol.aat6975 30341145

[48] Jeon SG, Kayama H, Ueda Y, Takahashi T, Asahara T, Tsuji H, et al Probiotic Bifidobacterium breve induces IL-10-producing Tr1 cells in the colon. PLoS Pathog 2012, 8, e1002714. 22693446 10.1371/journal.ppat.1002714 PMC3364948

[49] Yang BH, Hagemann S, Mamareli P, Lauer U, Hoffmann U, Beckstette M, et al Foxp3(+) T cells expressing RORγt represent a stable regulatory T-cell effector lineage with enhanced suppressive capacity during intestinal inflammation. Mucosal Immunol 2016, 9, 444–57. 26307665 10.1038/mi.2015.74

[50] Ohnmacht C, Park JH, Cording S, Wing JB, Atarashi K, Obata Y, et al MUCOSAL IMMUNOLOGY. The microbiota regulates type 2 immunity through RORγt+ T cells. Science 2015, 349, 989–93. 26160380 10.1126/science.aac4263

[51] Thornton AM, Korty PE, Tran DQ, Wohlfert EA, Murray PE, Belkaid Y, et al Expression of Helios, an Ikaros transcription factor family member, differentiates thymic-derived from peripherally induced Foxp3+ T regulatory cells. J Immunol 2010, 184, 3433–41. 20181882 10.4049/jimmunol.0904028 PMC3725574

[52] Weiss JM, Bilate AM, Gobert M, Ding Y, Curotto de Lafaille MA, Parkhurst CN, et al Neuropilin 1 is expressed on thymus-derived natural regulatory T cells, but not mucosa-generated induced Foxp3+ T reg cells. J Exp Med 2012, 209, 1723–42, S1. 22966001 10.1084/jem.20120914 PMC3457733

[53] Yadav M, Louvet C, Davini D, Gardner JM, Martinez-Llordella M, Bailey-Bucktrout S, et al Neuropilin-1 distinguishes natural and inducible regulatory T cells among regulatory T cell subsets *in vivo*. J Exp Med 2012, 209, 1713–22, S1–19. 22966003 10.1084/jem.20120822 PMC3457729

[54] Gottschalk RA, Corse E, Allison JP. Expression of Helios in peripherally induced Foxp3+ regulatory T cells. J Immunol 2012, 188, 976–80. 22198953 10.4049/jimmunol.1102964

[55] Akimova T, Beier UH, Wang L, Levine MH, Hancock WW. Helios expression is a marker of T cell activation and proliferation. PLoS One 2011, 6, e24226. 21918685 10.1371/journal.pone.0024226 PMC3168881

[56] Szurek E, Cebula A, Wojciech L, Pietrzak M, Rempala G, Kisielow P, et al Differences in expression level of Helios and neuropilin-1 do not distinguish thymus-derived from extrathymically-induced CD4+Foxp3+ regulatory T cells. PLoS One 2015, 10, e0141161. 26495986 10.1371/journal.pone.0141161 PMC4619666

[57] Chi X, Wang CH, Parisotto YF, Nyberg WA, Cabric V, Gelineau A, et al Decoding Peripheral Tolerance: TCR rules for pTreg differentiation in the Gut. bioRxiv 683415, 10.1101/2025.10.20.683415, 20 October 2025, preprint: not peer reviewed.

[58] Pratama A, Schnell A, Mathis D, Benoist C. Developmental and cellular age direct conversion of CD4+ T cells into RORγ+ or Helios+ colon Treg cells. J Exp Med 2020, 217, e20190428. 31685531 10.1084/jem.20190428 PMC7037252

[59] Ramanan D, Chowdhary K, Candéias SM, Sassone-Corsi M, Gelineau A, Mathis D, et al Homeostatic, repertoire and transcriptional relationships between colon T regulatory cell subsets. Proc Natl Acad Sci U S A 2023, 120, e2311566120. 38064511 10.1073/pnas.2311566120 PMC10723124

[60] Solomon BD, Hsieh CS. Antigen-specific development of mucosal Foxp3+RORγt+ T cells from regulatory T cell precursors. J Immunol 2016, 197, 3512–9. 27671109 10.4049/jimmunol.1601217 PMC5101183

[61] Bhaumik S, Mickael ME, Moran M, Spell M, Basu R. RORγt promotes Foxp3 expression by antagonizing the effector program in colonic regulatory T cells. J Immunol 2021, 207, 2027–38. 34518282 10.4049/jimmunol.2100175 PMC8490938

[62] Haribhai D, Lin W, Edwards B, Ziegelbauer J, Salzman NH, Carlson MR, et al A central role for induced regulatory T cells in tolerance induction in experimental colitis. J Immunol 2009, 182, 3461–8. 19265124 10.4049/jimmunol.0802535 PMC2763205

[63] Chudnovskiy A, Mortha A, Kana V, Kennard A, Ramirez JD, Rahman A, et al Host-Protozoan Interactions Protect from Mucosal Infections through Activation of the Inflammasome. Cell 2016, 167, 444–456 e14. 27716507 10.1016/j.cell.2016.08.076 PMC5129837

[64] Zegarra-Ruiz DF, Kim DV, Norwood K, Kim M, Wu WH, Saldana-Morales FB, et al Thymic development of gut-microbiota-specific T cells. Nature 2021, 594, 413–7. 33981034 10.1038/s41586-021-03531-1 PMC8323488

[65] Geem D, Medina-Contreras O, McBride M, Newberry RD, Koni PA, Denning TL. Specific microbiota-induced intestinal Th17 differentiation requires MHC class II but not GALT and mesenteric lymph nodes. J Immunol 2014, 193, 431–8. 24899505 10.4049/jimmunol.1303167 PMC4097179

[66] Goto Y, Panea C, Nakato G, Cebula A, Lee C, Diez MG, et al Segmented filamentous bacteria antigens presented by intestinal dendritic cells drive mucosal Th17 cell differentiation. Immunity 2014, 40, 594–607. 24684957 10.1016/j.immuni.2014.03.005 PMC4084624

[67] Matzinger P . Friendly and dangerous signals: is the tissue in control? Nat Immunol 2007, 8, 11–3. 17179963 10.1038/ni0107-11

[68] Arpaia N, Green JA, Moltedo B, Arvey A, Hemmers S, Yuan S, et al A distinct function of regulatory T cells in tissue protection. Cell 2015, 162, 1078–89. 26317471 10.1016/j.cell.2015.08.021 PMC4603556

[69] Loffredo LF, Kaiser KA, Kornberg A, Rao S, de Los Santos-Alexis K, Han A, et al An amphiregulin reporter mouse enables transcriptional and clonal expansion analysis of reparative lung Tregs. JCI Insight 2025, 10, e187245. 40626358 10.1172/jci.insight.187245 PMC12288904

[70] Wang L, Wang S, Lin J, Li J, Wang M, Yu J, et al Treg and intestinal myofibroblasts-derived Amphiregulin induced by TGF-β mediates intestinal fibrosis in Crohn’s disease. J Transl Med 2025, 23, 452. 40247299 10.1186/s12967-025-06413-6 PMC12004752

[71] Zhao X, Yang W, Yu T, Yu Y, Cui X, Zhou Z, et al Th17 cell-derived amphiregulin promotes colitis-associated intestinal fibrosis through activation of mTOR and MEK in intestinal myofibroblasts. Gastroenterology 2023, 164, 89–102. 36113570 10.1053/j.gastro.2022.09.006 PMC9772145

[72] Ansaldo E, Slayden LC, Ching KL, Koch MA, Wolf NK, Plichta DR, et al *Akkermansia muciniphila* induces intestinal adaptive immune responses during homeostasis. Science 2019, 364, 1179–84. 31221858 10.1126/science.aaw7479 PMC6645389

[73] Pabst O, Slack E. Iga and the intestinal microbiota: the importance of being specific. Mucosal Immunol 2020, 13, 12–21. 31740744 10.1038/s41385-019-0227-4 PMC6914667

[74] Pabst O . New concepts in the generation and functions of IgA. Nat Rev Immunol 2012, 12, 821–32. 23103985 10.1038/nri3322

[75] Kau AL, Planer JD, Liu J, Rao S, Yatsunenko T, Trehan I, et al Functional characterization of IgA-targeted bacterial taxa from undernourished Malawian children that produce diet-dependent enteropathy. Sci Transl Med 2015, 7, 276ra24. 10.1126/scitranslmed.aaa4877 PMC4423598 25717097

[76] Palm NW, de Zoete MR, Cullen TW, Barry NA, Stefanowski J, Hao L, et al Immunoglobulin A coating identifies colitogenic bacteria in inflammatory bowel disease. Cell 2014, 158, 1000–10. 25171403 10.1016/j.cell.2014.08.006 PMC4174347

[77] Macpherson AJ, Gatto D, Sainsbury E, Harriman GR, Hengartner H, Zinkernagel RM. A primitive T cell-independent mechanism of intestinal mucosal IgA responses to commensal bacteria. Science 2000, 288, 2222–6. 10864873 10.1126/science.288.5474.2222

[78] Kawamoto S, Maruya M, Kato LM, Suda W, Atarashi K, Doi Y, et al Foxp3(+) T cells regulate immunoglobulin a selection and facilitate diversification of bacterial species responsible for immune homeostasis. Immunity 2014, 41, 152–65. 25017466 10.1016/j.immuni.2014.05.016

[79] Kubinak JL, Petersen C, Stephens WZ, Soto R, Bake E, O'Connell RM, et al Myd88 signaling in T cells directs IgA-mediated control of the microbiota to promote health. Cell Host Microbe 2015, 17, 153–63. 25620548 10.1016/j.chom.2014.12.009 PMC4451207

[80] Cong Y, Feng T, Fujihashi K, Schoeb TR, Elson CO. A dominant, coordinated T regulatory cell-IgA response to the intestinal microbiota. Proc Natl Acad Sci U S A 2009, 106, 19256–61. 19889972 10.1073/pnas.0812681106 PMC2780781

[81] Cao AT, Yao S, Gong B, Nurieva RI, Elson CO, Cong Y. Interleukin (IL)-21 promotes intestinal IgA response to microbiota. Mucosal Immunol 2015, 8, 1072–82. 25586558 10.1038/mi.2014.134 PMC4501922

[82] Hirota K, Turner JE, Villa M, Duarte JH, Demengeot J, Steinmetz OM, et al Plasticity of Th17 cells in Peyer's patches is responsible for the induction of T cell-dependent IgA responses. Nat Immunol 2013, 14, 372–9. 23475182 10.1038/ni.2552 PMC3672955

[83] Tsuji M, Komatsu N, Kawamoto S, Suzuki K, Kanagawa O, Honjo T, et al Preferential generation of follicular B helper T cells from Foxp3+ T cells in gut Peyer's patches. Science 2009, 323, 1488–92. 19286559 10.1126/science.1169152

[84] Sutherland DB, Suzuki K, Fagarasan S. Fostering of advanced mutualism with gut microbiota by immunoglobulin A. Immunol Rev 2016, 270, 20–31. 26864102 10.1111/imr.12384

[85] Kulkarni DH, Newberry RD. Antigen uptake in the gut: an underappreciated piece to the puzzle? Annu Rev Immunol 2025, 43, 571–88. 40279313 10.1146/annurev-immunol-082523-090154 PMC12068241

[86] Komban RJ, Strömberg A, Biram A, Cervin J, Lebrero-Fernández C, Mabbott N, et al Activated Peyer's patch B cells sample antigen directly from M cells in the subepithelial dome. Nat Commun 2019, 10, 2423. 31160559 10.1038/s41467-019-10144-w PMC6547658

[87] Lelouard H, Fallet M, de Bovis B, Méresse S, Gorvel JP. Peyer's patch dendritic cells sample antigens by extending dendrites through M cell-specific transcellular pores. Gastroenterology 2012, 142, 592–601.e3. 22155637 10.1053/j.gastro.2011.11.039

[88] Lelouard H, Henri S, De Bovis B, Mugnier B, Chollat-Namy A, Malissen B, et al Pathogenic bacteria and dead cells are internalized by a unique subset of Peyer's patch dendritic cells that express lysozyme. Gastroenterology 2010, 138, 173–84.e1–3. 19800337 10.1053/j.gastro.2009.09.051

[89] Rios D, Wood MB, Li J, Chassaing B, Gewirtz AT, Williams IR. Antigen sampling by intestinal M cells is the principal pathway initiating mucosal IgA production to commensal enteric bacteria. Mucosal Immunol 2016, 9, 907–16. 26601902 10.1038/mi.2015.121 PMC4917673

[90] Corr SC, Gahan CC, Hill C. M-cells: origin, morphology and role in mucosal immunity and microbial pathogenesis. FEMS Immunol Med Microbiol 2008, 52, 2–12. 18081850 10.1111/j.1574-695X.2007.00359.x

[91] Lapthorne S, Macsharry J, Scully P, Nally K, Shanahan F. Differential intestinal M-cell gene expression response to gut commensals. Immunology 2012, 136, 312–24. 22385384 10.1111/j.1365-2567.2012.03581.x PMC3385031

[92] Lee AY, Chang SY, Kim JI, Cha HR, Jang MH, Yamamoto M, et al Dendritic cells in colonic patches and iliac lymph nodes are essential in mucosal IgA induction following intrarectal administration via CCR7 interaction. Eur J Immunol 2008, 38, 1127–37. 18350542 10.1002/eji.200737442

[93] Masahata K, Umemoto E, Kayama H, Kotani M, Nakamura S, Kurakawa T, et al Generation of colonic IgA-secreting cells in the caecal patch. Nat Commun 2014, 5, 3704. 24718324 10.1038/ncomms4704

[94] Agace WW, McCoy KD. Regionalized development and maintenance of the intestinal adaptive immune landscape. Immunity 2017, 46, 532–48. 28423335 10.1016/j.immuni.2017.04.004

[95] Newberry RD, Lorenz RG. Organizing a mucosal defense. Immunol Rev 2005, 206, 6–21. 16048539 10.1111/j.0105-2896.2005.00282.x

[96] Owen RL, Piazza AJ, Ermak TH. Ultrastructural and cytoarchitectural features of lymphoreticular organs in the colon and rectum of adult BALB/c mice. Am J Anat 1991, 190, 10–8. 1984672 10.1002/aja.1001900103

[97] Lorenz RG, Newberry RD. Isolated lymphoid follicles can function as sites for induction of mucosal immune responses. Ann N Y Acad Sci 2004, 1029, 44–57. 15681742 10.1196/annals.1309.006

[98] Lorenz RG, Chaplin DD, McDonald KG, McDonough JS, Newberry RD. Isolated lymphoid follicle formation is inducible and dependent upon lymphotoxin-sufficient B lymphocytes, lymphotoxin beta receptor, and TNF receptor I function. J Immunol 2003, 170, 5475–82. 12759424 10.4049/jimmunol.170.11.5475

[99] Pabst O, Herbrand H, Friedrichsen M, Velaga S, Dorsch M, Berhardt G, et al Adaptation of solitary intestinal lymphoid tissue in response to microbiota and chemokine receptor CCR7 signaling. J Immunol 2006, 177, 6824–32. 17082596 10.4049/jimmunol.177.10.6824

[100] Lecuyer E, Rakotobe S, Lengliné-Garnier H, Lebreton C, Picard M, Juste C, et al Segmented filamentous bacterium uses secondary and tertiary lymphoid tissues to induce gut IgA and specific T helper 17 cell responses. Immunity 2014, 40, 608–20. 24745335 10.1016/j.immuni.2014.03.009

[101] Pabst O, Herbrand H, Worbs T, Friedrichsen M, Yan S, Hoffmann MW, et al Cryptopatches and isolated lymphoid follicles: dynamic lymphoid tissues dispensable for the generation of intraepithelial lymphocytes. Eur J Immunol 2005, 35, 98–107. 15580658 10.1002/eji.200425432

[102] Baptista AP, Olivier BJ, Goverse G, Greuter M, Knippenberg M, Kusser K, et al Colonic patch and colonic SILT development are independent and differentially regulated events. Mucosal Immunol 2013, 6, 511–21. 22990625 10.1038/mi.2012.90 PMC3570605

[103] Knoop KA, Butler BR, Kumar N, Newberry RD, Williams IR. Distinct developmental requirements for isolated lymphoid follicle formation in the small and large intestine: RANKL is essential only in the small intestine. Am J Pathol 2011, 179, 1861–71. 21854748 10.1016/j.ajpath.2011.06.004 PMC3181393

[104] Knoop KA, Newberry RD. Isolated lymphoid follicles are dynamic reservoirs for the induction of intestinal IgA. Front Immunol 2012, 3, 84. 22566964 10.3389/fimmu.2012.00084 PMC3343265

[105] Fenton TM, Jørgensen PB, Niss K, Rubin SJS, Mörbe UM, Riis LB, et al Immune profiling of human gut-associated lymphoid tissue identifies a role for isolated lymphoid follicles in priming of region-specific immunity. Immunity 2020, 52, 557–70.e6. 32160523 10.1016/j.immuni.2020.02.001 PMC7155934

[106] Senda T, Dogra P, Granot T, Furuhashi K, Snyder ME, Carpenter DJ, et al Microanatomical dissection of human intestinal T-cell immunity reveals site-specific changes in gut-associated lymphoid tissues over life. Mucosal Immunol 2019, 12, 378–89. 30523311 10.1038/s41385-018-0110-8 PMC6375790

[107] Rescigno M, Urbano M, Valzasina B, Francolini M, Rotta G, Bonasio R, et al Dendritic cells express tight junction proteins and penetrate gut epithelial monolayers to sample bacteria. Nat Immunol 2001, 2, 361–7. 11276208 10.1038/86373

[108] Rescigno M, Rotta G, Valzasina B, Ricciardi-Castagnoli P. Dendritic cells shuttle microbes across gut epithelial monolayers. Immunobiology 2001, 204, 572–81. 11846220 10.1078/0171-2985-00094

[109] Chieppa M, Rescigno M, Huang AY, Germain RN. Dynamic imaging of dendritic cell extension into the small bowel lumen in response to epithelial cell TLR engagement. J Exp Med 2006, 203, 2841–52. 17145958 10.1084/jem.20061884 PMC2118178

[110] Niess JH, Brand S, Gu X, Landsman L, Jung S, McCormick BA, et al CX3CR1-mediated dendritic cell access to the intestinal lumen and bacterial clearance. Science 2005, 307, 254–8. 15653504 10.1126/science.1102901

[111] Kulkarni DH, Gustafsson JK, Knoop KA, McDonald KG, Bidani SS, Davis JE, et al Goblet cell associated antigen passages support the induction and maintenance of oral tolerance. Mucosal Immunol 2020, 13, 271–82. 31819172 10.1038/s41385-019-0240-7 PMC7044050

[112] McDole JR, Wheeler LW, McDonald KG, Wang B, Konjufca V, Knoop KA, et al Goblet cells deliver luminal antigen to CD103+ dendritic cells in the small intestine. Nature 2012, 483, 345–9. 22422267 10.1038/nature10863 PMC3313460

[113] Udayan S, Floyd AN, John V, Barrios BE, Rusconi BA, McDonald KG, et al Colonic goblet cell-associated antigen passages mediate physiologic and beneficial translocation of live gut bacteria in preweaning mice. Nat Microbiol 2025, 10, 927–38. 40169738 10.1038/s41564-025-01965-1 PMC12704251

[114] Knoop KA, McDonald KG, McCrate S, McDole JR, Newberry RD. Microbial sensing by goblet cells controls immune surveillance of luminal antigens in the colon. Mucosal Immunol 2015, 8, 198–210. 25005358 10.1038/mi.2014.58 PMC4268115

[115] Kulkarni DH, McDonald KG, Knoop KA, Gustafsson JK, Kozlowski KM, Hunstad DA, et al Goblet cell associated antigen passages are inhibited during Salmonella typhimurium infection to prevent pathogen dissemination and limit responses to dietary antigens. Mucosal Immunol 2018, 11, 1103–13. 29445136 10.1038/s41385-018-0007-6 PMC6037413

[116] Kaparakis-Liaskos M, Ferrero RL. Immune modulation by bacterial outer membrane vesicles. Nat Rev Immunol 2015, 15, 375–87. 25976515 10.1038/nri3837

[117] Brown L, Wolf JM, Prados-Rosales R, Casadevall A. Through the wall: extracellular vesicles in Gram-positive bacteria, mycobacteria and fungi. Nat Rev Microbiol 2015, 13, 620–30. 26324094 10.1038/nrmicro3480 PMC4860279

[118] Hickey CA, Kuhn KA, Donermeyer DL, Porter NT, Jin C, Cameron EA, et al Colitogenic bacteroides thetaiotaomicron antigens access host immune cells in a sulfatase-dependent manner via outer membrane vesicles. Cell Host Microbe 2015, 17, 672–80. 25974305 10.1016/j.chom.2015.04.002 PMC4432250

[119] Shen Y, Giardino Torchia ML, Lawson GW, Karp CL, Ashwell JD, Mazmanian SK. Outer membrane vesicles of a human commensal mediate immune regulation and disease protection. Cell Host Microbe 2012, 12, 509–20. 22999859 10.1016/j.chom.2012.08.004 PMC3895402

[120] Engelhart MJ, Glowacki RWP, Till JM, Harding CV, Martens EC, Ahern PP. The NQR complex regulates the immunomodulatory function of bacteroides thetaiotaomicron. J Immunol 2023, 211, 767–81. 37486212 10.4049/jimmunol.2200892 PMC10527448

[121] Durant L, Stentz R, Noble A, Brooks J, Gicheva N, Reddi D, et al Bacteroides thetaiotaomicron-derived outer membrane vesicles promote regulatory dendritic cell responses in health but not in inflammatory bowel disease. Microbiome 2020, 8, 88. 32513301 10.1186/s40168-020-00868-z PMC7282036

[122] Fonseca S, Carvalho AL, Miquel-Clopés A, Jones EJ, Juodeikis R, Stentz R, et al Extracellular vesicles produced by the human gut commensal bacterium Bacteroides thetaiotaomicron elicit anti-inflammatory responses from innate immune cells. Front Microbiol 2022, 13, 1050271. 36439842 10.3389/fmicb.2022.1050271 PMC9684339

[123] Rodriguez BV, Kuehn MJ. Staphylococcus aureus secretes immunomodulatory RNA and DNA via membrane vesicles. Sci Rep 2020, 10, 18293. 33106559 10.1038/s41598-020-75108-3 PMC7589478

[124] Ladinsky MS, Araujo LP, Zhang X, Veltri J, Galan-Diez M, Soualhi S, et al Endocytosis of commensal antigens by intestinal epithelial cells regulates mucosal T cell homeostasis. Science 2019, 363, eaat4042. 10.1126/science.aat4042 PMC6708280 30846568

[125] Mazmanian SK, Round JL, Kasper DL. A microbial symbiosis factor prevents intestinal inflammatory disease. Nature 2008, 453, 620–5. 18509436 10.1038/nature07008

[126] Macpherson AJ, Uhr T. Induction of protective IgA by intestinal dendritic cells carrying commensal bacteria. Science 2004, 303, 1662–5. 15016999 10.1126/science.1091334

[127] Meyerholz DK, Stabel TJ, Cheville NF. Segmented filamentous bacteria interact with intraepithelial mononuclear cells. Infect Immun 2002, 70, 3277–80. 12011024 10.1128/IAI.70.6.3277-3280.2002 PMC128008

[128] Umesaki Y, Setoyama H, Matsumoto S, Imaoka A, Itoh K. Differential roles of segmented filamentous bacteria and clostridia in development of the intestinal immune system. Infect Immun 1999, 67, 3504–11. 10377132 10.1128/iai.67.7.3504-3511.1999 PMC116537

[129] Jepson MA, Clark MA, Simmons NL, Hirst BH. Actin accumulation at sites of attachment of indigenous apathogenic segmented filamentous bacteria to mouse ileal epithelial cells. Infect Immun 1993, 61, 4001–4. 8359925 10.1128/iai.61.9.4001-4004.1993 PMC281108

[130] Koopman JP, Stadhouders AM, Kennis HM, De Boer H. The attachment of filamentous segmented micro-organisms to the distal ileum wall of the mouse: a scanning and transmission electron microscopy study. Lab Anim 1987, 21, 48–52. 3560864 10.1258/002367787780740743

[131] Yamauchi KE, Snel J. Transmission electron microscopic demonstration of phagocytosis and intracellular processing of segmented filamentous bacteria by intestinal epithelial cells of the chick ileum. Infect Immun 2000, 68, 6496–504. 11035767 10.1128/iai.68.11.6496-6504.2000 PMC97741

[132] Sczesnak A, Segata N, Qin X, Gevers D, Petrosino JF, Huttenhower C, et al The genome of th17 cell-inducing segmented filamentous bacteria reveals extensive auxotrophy and adaptations to the intestinal environment. Cell Host Microbe 2011, 10, 260–72. 21925113 10.1016/j.chom.2011.08.005 PMC3209701

[133] Chase DG, Erlandsen SL. Evidence for a complex life cycle and endospore formation in the attached, filamentous, segmented bacterium from murine ileum. J Bacteriol 1976, 127, 572–83. 931952 10.1128/jb.127.1.572-583.1976 PMC233091

[134] Blumershine RV, Savage DC. Filamentous microbes indigenous to the murine small bowel: a scanning electron microscopic study of their morphology and attachment to the epithelium. Microb Ecol 1977, 4, 95–103. 24231968 10.1007/BF02014280

[135] Atarashi K, Tanoue T, Ando M, Kamada N, Nagano Y, Narushima S, et al Th17 cell induction by adhesion of microbes to intestinal epithelial cells. Cell 2015, 163, 367–80. 26411289 10.1016/j.cell.2015.08.058 PMC4765954

[136] Tannock GW, Miller JR, Savage DC. Host specificity of filamentous, segmented microorganisms adherent to the small bowel epithelium in mice and rats. Appl Environ Microbiol 1984, 47, 441–2. 6712214 10.1128/aem.47.2.441-442.1984 PMC239693

[137] Sano T, Huang W, Hall JA, Yang Y, Chen A, Gavzy SJ, et al An IL-23R/IL-22 circuit regulates epithelial serum amyloid A to promote local effector Th17 responses. Cell 2015, 163, 381–93. 26411290 10.1016/j.cell.2015.08.061 PMC4621768

[138] Hayashi A, Sato T, Kamada N, Mikami Y, Matsuoka K, Hisamatsu T, et al A single strain of Clostridium butyricum induces intestinal IL-10-producing macrophages to suppress acute experimental colitis in mice. Cell Host Microbe 2013, 13, 711–22. 23768495 10.1016/j.chom.2013.05.013

[139] Donaldson GP, Chou WC, Manson AL, Rogov P, Abeel T, Bochicchio J, et al Spatially distinct physiology of Bacteroides fragilis within the proximal colon of gnotobiotic mice. Nat Microbiol 2020, 5, 746–56. 32152589 10.1038/s41564-020-0683-3 PMC7426998

[140] Huang JY, Lee SM, Mazmanian SK. The human commensal Bacteroides fragilis binds intestinal mucin. Anaerobe 2011, 17, 137–41. 21664470 10.1016/j.anaerobe.2011.05.017 PMC3163789

[141] Lee SM, Donaldson GP, Mikulski Z, Boyajian S, Ley K, Mazmanian SK. Bacterial colonization factors control specificity and stability of the gut microbiota. Nature 2013, 501, 426–9. 23955152 10.1038/nature12447 PMC3893107

[142] Nava GM, Friedrichsen HJ, Stappenbeck TS. Spatial organization of intestinal microbiota in the mouse ascending colon. ISME J 2011, 5, 627–38. 20981114 10.1038/ismej.2010.161 PMC3105732

[143] Kuehl CJ, Wood HD, Marsh TL, Schmidt TM, Young VB. Colonization of the cecal mucosa by Helicobacter hepaticus impacts the diversity of the indigenous microbiota. Infect Immun 2005, 73, 6952–61. 16177375 10.1128/IAI.73.10.6852-6961.2005 PMC1230902

[144] Fox JG, Li X, Yan L, Cahill RJ, Hurley R, Lewis R, et al Chronic proliferative hepatitis in A/JCr mice associated with persistent Helicobacter hepaticus infection: a model of helicobacter-induced carcinogenesis. Infect Immun 1996, 64, 1548–58. 8613359 10.1128/iai.64.5.1548-1558.1996 PMC173960

[145] Liu LM, MacPherson GG. Antigen acquisition by dendritic cells: intestinal dendritic cells acquire antigen administered orally and can prime naive T cells *in vivo*. J Exp Med 1993, 177, 1299–307. 8478609 10.1084/jem.177.5.1299 PMC2191013

[146] Steinman RM . The dendritic cell system and its role in immunogenicity. Annu Rev Immunol 1991, 9, 271–96. 1910679 10.1146/annurev.iy.09.040191.001415

[147] MacPherson GG, Jenkins CD, Stein MJ, Edwards C. Endotoxin-mediated dendritic cell release from the intestine. Characterization of released dendritic cells and TNF dependence. J Immunol 1995, 154, 1317–22. 7822800

[148] Bogunovic M, Ginhoux F, Helft J, Shang L, Hashimoto D, Greter M, et al Origin of the lamina propria dendritic cell network. Immunity 2009, 31, 513–25. 19733489 10.1016/j.immuni.2009.08.010 PMC2778256

[149] Uematsu S, Fujimoto K, Jang MH, Yang BG, Jung YJ, Nishiyama M, et al Regulation of humoral and cellular gut immunity by lamina propria dendritic cells expressing Toll-like receptor 5. Nat Immunol 2008, 9, 769–76. 18516037 10.1038/ni.1622

[150] Coombes JL, Siddiqui KR, Arancibia-Cárcamo CV, Hall J, Sun CM, Belkaid Y, et al A functionally specialized population of mucosal CD103+ DCs induces Foxp3+ regulatory T cells via a TGF-beta and retinoic acid-dependent mechanism. J Exp Med 2007, 204, 1757–64. 17620361 10.1084/jem.20070590 PMC2118683

[151] Sun CM, Hall JA, Blank RB, Bouladoux N, Oukka M, Mora JR, et al Small intestine lamina propria dendritic cells promote *de novo* generation of Foxp3 T reg cells via retinoic acid. J Exp Med 2007, 204, 1775–85. 17620362 10.1084/jem.20070602 PMC2118682

[152] Cerovic V, Houston SA, Scott CL, Aumeunier A, Yrlid U, Mowat AM, et al Intestinal CD103(-) dendritic cells migrate in lymph and prime effector T cells. Mucosal Immunol 2013, 6, 104–13. 22718260 10.1038/mi.2012.53

[153] Lewis KL, Caton ML, Bogunovic M, Greter M, Grajkowska LT, Ng D, et al Notch2 receptor signaling controls functional differentiation of dendritic cells in the spleen and intestine. Immunity 2011, 35, 780–91. 22018469 10.1016/j.immuni.2011.08.013 PMC3225703

[154] Schlitzer A, McGovern N, Teo P, Zelante T, Atarashi K, Low D, et al IRF4 transcription factor-dependent CD11b+ dendritic cells in human and mouse control mucosal IL-17 cytokine responses. Immunity 2013, 38, 970–83. 23706669 10.1016/j.immuni.2013.04.011 PMC3666057

[155] Persson EK, Uronen-Hansson H, Semmrich M, Rivollier A, Hägerbrand K, Marsal J, et al IRF4 transcription-factor-dependent CD103(+)CD11b(+) dendritic cells drive mucosal T helper 17 cell differentiation. Immunity 2013, 38, 958–69. 23664832 10.1016/j.immuni.2013.03.009

[156] Welty NE, Staley C, Ghilardi N, Sadowsky MJ, Igyártó BZ, Kaplan DH. Intestinal lamina propria dendritic cells maintain T cell homeostasis but do not affect commensalism. J Exp Med 2013, 210, 2011–24. 24019552 10.1084/jem.20130728 PMC3782055

[157] McKenna HJ, Stocking KL, Miller RE, Brasel K, De Smedt T, Maraskovsky E, et al Mice lacking flt3 ligand have deficient hematopoiesis affecting hematopoietic progenitor cells, dendritic cells, and natural killer cells. Blood 2000, 95, 3489–97. 10828034

[158] Panea C, Farkas AM, Goto Y, Abdollahi-Roodsaz S, Lee C, Koscsó B, et al Intestinal monocyte-derived macrophages control commensal-specific Th17 responses. Cell Rep 2015, 12, 1314–24. 26279572 10.1016/j.celrep.2015.07.040 PMC4567384

[159] Banks TA, Rouse BT, Kerley MK, Blair PJ, Godfrey VL, Kuklin NA, et al Lymphotoxin-alpha-deficient mice. Effects on secondary lymphoid organ development and humoral immune responsiveness. J Immunol 1995, 155, 1685–93. 7636227

[160] Schulz O, Jaensson E, Persson EK, Liu X, Worbs T, Agace WW, et al Intestinal CD103+, but not CX3CR1+, antigen sampling cells migrate in lymph and serve classical dendritic cell functions. J Exp Med 2009, 206, 3101–14. 20008524 10.1084/jem.20091925 PMC2806467

[161] Sano T, Kageyama T, Fang V, Kedmi R, Martinez CS, Talbot J, et al Redundant cytokine requirement for intestinal microbiota-induced Th17 cell differentiation in draining lymph nodes. Cell Rep 2021, 36, 109608. 34433045 10.1016/j.celrep.2021.109608 PMC8845566

[162] Ngoi S, Yang Y, Iwanowycz S, Gutierrez J, Li Y, Williams C, et al Migrating type 2 dendritic cells prime mucosal Th17 cells specific to small intestinal commensal bacteria. J Immunol 2022, 209, 1200–11. 35995508 10.4049/jimmunol.2200204 PMC9492644

[163] Meredith MM, Liu K, Darrasse-Jeze G, Kamphorst AO, Schreiber HA, Guermonprez P, et al Expression of the zinc finger transcription factor zDC (Zbtb46, Btbd4) defines the classical dendritic cell lineage. J Exp Med 2012, 209, 1153–65. 22615130 10.1084/jem.20112675 PMC3371731

[164] Mazzini E, Massimiliano L, Penna G, Rescigno M. Oral tolerance can be established via gap junction transfer of fed antigens from CX3CR1⁺ macrophages to CD103⁺ dendritic cells. Immunity 2014, 40, 248–61. 24462723 10.1016/j.immuni.2013.12.012

[165] Russler-Germain EV, Yi J, Young S, Nutsch K, Wong HS, Ai TL, et al Gut Helicobacter presentation by multiple dendritic cell subsets enables context-specific regulatory T cell generation. Elife 2021, 10, e54792. 33533717 10.7554/eLife.54792 PMC7877908

[166] Gu Y, Bartolomé-Casado R, Xu C, Bertocchi A, Janney A, Heuberger C, et al Immune microniches shape intestinal T(reg) function. Nature 2024, 628, 854–62. 38570678 10.1038/s41586-024-07251-0 PMC11041794

[167] Kedmi R, Najar TA, Mesa KR, Grayson A, Kroehling L, Hao Y, et al A RORγt(+) cell instructs gut microbiota-specific T(reg) cell differentiation. Nature 2022, 610, 737–43. 36071167 10.1038/s41586-022-05089-y PMC9908423

[168] Hepworth MR, Monticelli LA, Fung TC, Ziegler CG, Grunberg S, Sinha R, et al Innate lymphoid cells regulate CD4+ T-cell responses to intestinal commensal bacteria. Nature 2013, 498, 113–7. 23698371 10.1038/nature12240 PMC3699860

[169] Lyu M, Suzuki H, Kang L, Gaspal F, Zhou W, Goc J, et al ILC3s select microbiota-specific regulatory T cells to establish tolerance in the gut. Nature 2022, 610, 744–51. 36071169 10.1038/s41586-022-05141-x PMC9613541

[170] Akagbosu B, Tayyebi Z, Shibu G, Paucar Iza YA, Deep D, Parisotto YF, et al Novel antigen-presenting cell imparts T(reg)-dependent tolerance to gut microbiota. Nature 2022, 610, 752–60. 36070798 10.1038/s41586-022-05309-5 PMC9605865

[171] Sun IH, Qualls AE, Yin HS, Wang J, Arvedson MP, Germino J, et al RORγt eTACs mediate oral tolerance and Treg induction. J Exp Med 2025, 222, e20250573. 40298935 10.1084/jem.20250573 PMC12039581

[172] Wang J, Lareau CA, Bautista JL, Gupta AR, Sandor K, Germino J, et al Single-cell multiomics defines tolerogenic extrathymic Aire-expressing populations with unique homology to thymic epithelium. Sci Immunol 2021, 6, eabl5053. 34767455 10.1126/sciimmunol.abl5053 PMC8855935

[173] Fu L, Upadhyay R, Pokrovskii M, Chen FM, Romero-Meza G, Griesemer A, et al PRDM16-dependent antigen-presenting cells induce tolerance to gut antigens. Nature 2025, 642, 756–65. 40228524 10.1038/s41586-025-08982-4 PMC12176658

[174] Rodrigues PF, Wu S, Trsan T, Panda SK, Fachi JL, Liu Y, et al Rorgammat-positive dendritic cells are required for the induction of peripheral regulatory T cells in response to oral antigens. Cell 2025, 188, 2720–37.e22. 40185101 10.1016/j.cell.2025.03.020 PMC12085292

[175] Schraml BU, van Blijswijk J, Zelenay S, Whitney PG, Filby A, Acton SE, et al Genetic tracing via DNGR-1 expression history defines dendritic cells as a hematopoietic lineage. Cell 2013, 154, 843–58. 23953115 10.1016/j.cell.2013.07.014

[176] Cabric V, Franco Parisotto Y, Park T, Akagbosu B, Zhao Z, Lo Y, et al A wave of Thetis cells imparts tolerance to food antigens early in life. Science 2025, 389, 268–74. 40373113 10.1126/science.adp0535 PMC13245555

[177] Esterházy D, Loschko J, London M, Jove V, Oliveira TY, Mucida D. Classical dendritic cells are required for dietary antigen-mediated induction of peripheral T(reg) cells and tolerance. Nat Immunol 2016, 17, 545–55. 27019226 10.1038/ni.3408 PMC4837106

[178] Rudnitsky A, Oh H, Margolin M, Dassa B, Shteinberg I, Stoler-Barak L, et al A coordinated cellular network regulates tolerance to food. Nature 2025, 644, 231–40. 40425043 10.1038/s41586-025-09173-x PMC12328219

[179] Chen W, Jin W, Hardegen N, Lei KJ, Li L, Marinos N, et al Conversion of peripheral CD4+CD25- naive T cells to CD4+CD25+ regulatory T cells by TGF-beta induction of transcription factor Foxp3. J Exp Med 2003, 198, 1875–86. 14676299 10.1084/jem.20030152 PMC2194145

[180] Gorelik L, Flavell RA. Abrogation of TGFbeta signaling in T cells leads to spontaneous T cell differentiation and autoimmune disease. Immunity 2000, 12, 171–81. 10714683 10.1016/s1074-7613(00)80170-3

[181] Li MO, Sanjabi S, Flavell RA. Transforming growth factor-beta controls development, homeostasis, and tolerance of T cells by regulatory T cell-dependent and -independent mechanisms. Immunity 2006, 25, 455–71. 16973386 10.1016/j.immuni.2006.07.011

[182] Bain CC, Montgomery J, Scott CL, Kel JM, Girard-Madoux MJH, Martens L, et al TGFβR signalling controls CD103(+)CD11b(+) dendritic cell development in the intestine. Nat Commun 2017, 8, 620. 28931816 10.1038/s41467-017-00658-6 PMC5607002

[183] Atarashi K, Nishimura J, Shima T, Umesaki Y, Yamamoto M, Onoue M, et al ATP drives lamina propria T(H)17 cell differentiation. Nature 2008, 455, 808–12. 18716618 10.1038/nature07240

[184] Hepworth MR, Fung TC, Masur SH, Kelsen JR, McConnell FM, Dubrot J, et al Immune tolerance. Group 3 innate lymphoid cells mediate intestinal selection of commensal bacteria-specific CD4(+) T cells. Science 2015, 348, 1031–5. 25908663 10.1126/science.aaa4812 PMC4449822

[185] Hsieh CS, Macatonia SE, Tripp CS, Wolf SF, O'Garra A, Murphy KM. Development of TH1 CD4+ T cells through IL-12 produced by Listeria-induced macrophages. Science 1993, 260, 547–9. 8097338 10.1126/science.8097338

[186] Pantosti A, Tzianabos AO, Onderdonk AB, Kasper DL. Immunochemical characterization of two surface polysaccharides of Bacteroides fragilis. Infect Immun 1991, 59, 2075–82. 2037368 10.1128/iai.59.6.2075-2082.1991 PMC257968

[187] Tzianabos AO, Onderdonk AB, Rosner B, Cisneros RL, Kasper DL. Structural features of polysaccharides that induce intra-abdominal abscesses. Science 1993, 262, 416–9. 8211161 10.1126/science.8211161

[188] Dasgupta S, Erturk-Hasdemir D, Ochoa-Reparaz J, Reinecker HC, Kasper DL. Plasmacytoid dendritic cells mediate anti-inflammatory responses to a gut commensal molecule via both innate and adaptive mechanisms. Cell Host Microbe 2014, 15, 413–23. 24721570 10.1016/j.chom.2014.03.006 PMC4020153

[189] Mazmanian SK, Liu CH, Tzianabos AO, Kasper DL. An immunomodulatory molecule of symbiotic bacteria directs maturation of the host immune system. Cell 2005, 122, 107–18. 16009137 10.1016/j.cell.2005.05.007

[190] Erturk-Hasdemir D, Oh SF, Okan NA, Stefanetti G, Gazzaniga FS, Seeberger PH, et al Symbionts exploit complex signaling to educate the immune system. Proc Natl Acad Sci U S A 2019, 116, 26157–66. 31811024 10.1073/pnas.1915978116 PMC6936714

[191] Round JL, Lee SM, Li J, Tran G, Jabri B, Chatila TA, et al The Toll-like receptor 2 pathway establishes colonization by a commensal of the human microbiota. Science 2011, 332, 974–7. 21512004 10.1126/science.1206095 PMC3164325

[192] Wang Q, McLoughlin RM, Cobb BA, Charrel-Dennis M, Zaleski KJ, Golenbock D, et al A bacterial carbohydrate links innate and adaptive responses through Toll-like receptor 2. J Exp Med 2006, 203, 2853–63. 17178920 10.1084/jem.20062008 PMC2118167

[193] Danne C, Ryzhakov G, Martínez-López M, Ilott NE, Franchini F, Cuskin F, et al A large polysaccharide produced by helicobacter hepaticus induces an anti-inflammatory gene signature in macrophages. Cell Host Microbe 2017, 22, 733–45.e5. 29241040 10.1016/j.chom.2017.11.002 PMC5734933

[194] Henke MT, Brown EM, Cassilly CD, Vlamakis H, Xavier RJ, Clardy J. Capsular polysaccharide correlates with immune response to the human gut microbe *Ruminococcus gnavus*. Proc Natl Acad Sci U S A 2021, 118, e2007595118. 33972416 10.1073/pnas.2007595118 PMC8157926

[195] Hall JA, Bouladoux N, Sun CM, Wohlfert EA, Blank RB, Zhu Q, et al Commensal DNA limits regulatory T cell conversion and is a natural adjuvant of intestinal immune responses. Immunity 2008, 29, 637–49. 18835196 10.1016/j.immuni.2008.08.009 PMC2712925

[196] Bouladoux N, Hall JA, Grainger JR, dos Santos LM, Kann MG, Nagarajan V, et al Regulatory role of suppressive motifs from commensal DNA. Mucosal Immunol 2012, 5, 623–34. 22617839 10.1038/mi.2012.36 PMC3427718

[197] Wang Y, Yin Y, Chen X, Zhao Y, Wu Y, Li Y, et al Induction of intestinal Th17 cells by flagellins from segmented filamentous bacteria. Front Immunol 2019, 10, 2750. 31824516 10.3389/fimmu.2019.02750 PMC6883716

[198] Dessein R, Gironella M, Vignal C, Peyrin-Biroulet L, Sokol H, Secher T, et al Toll-like receptor 2 is critical for induction of Reg3 beta expression and intestinal clearance of Yersinia pseudotuberculosis. Gut 2009, 58, 771–6. 19174417 10.1136/gut.2008.168443

[199] Dziarski R, Gupta D. Staphylococcus aureus peptidoglycan is a toll-like receptor 2 activator: a reevaluation. Infect Immun 2005, 73, 5212–6. 16041042 10.1128/IAI.73.8.5212-5216.2005 PMC1201261

[200] Yang RB, Mark MR, Gray A, Huang A, Xie MH, Zhang M, et al Toll-like receptor-2 mediates lipopolysaccharide-induced cellular signalling. Nature 1998, 395, 284–8. 9751057 10.1038/26239

[201] Travassos LH, Girardin SE, Philpott DJ, Blanot D, Nahori MA, Werts C, et al Toll-like receptor 2-dependent bacterial sensing does not occur via peptidoglycan recognition. EMBO Rep 2004, 5, 1000–6. 15359270 10.1038/sj.embor.7400248 PMC1299148

[202] Høverstad T, Midtvedt T. Short-chain fatty acids in germfree mice and rats. J Nutr 1986, 116, 1772–6. 3761032 10.1093/jn/116.9.1772

[203] Mann ER, Lam YK, Uhlig HH. Short-chain fatty acids: linking diet, the microbiome and immunity. Nat Rev Immunol 2024, 24, 577–95. 38565643 10.1038/s41577-024-01014-8

[204] Smith PM, Howitt MR, Panikov N, Michaud M, Gallini CA, Bohlooly YM, et al The microbial metabolites, short-chain fatty acids, regulate colonic Treg cell homeostasis. Science 2013, 341, 569–73. 23828891 10.1126/science.1241165 PMC3807819

[205] Arpaia N, Campbell C, Fan X, Dikiy S, van der Veeken J, deRoos P, et al Metabolites produced by commensal bacteria promote peripheral regulatory T-cell generation. Nature 2013, 504, 451–5. 24226773 10.1038/nature12726 PMC3869884

[206] Furusawa Y, Obata Y, Fukuda S, Endo TA, Nakato G, Takahashi D, et al Commensal microbe-derived butyrate induces the differentiation of colonic regulatory T cells. Nature 2013, 504, 446–50. 24226770 10.1038/nature12721

[207] Oliver A, Alkan Z, Stephensen CB, Newman JW, Kable ME, Lemay DG. Diet, microbiome, and inflammation predictors of fecal and plasma short-chain fatty acids in humans. J Nutr 2024, 154, 3298–311. 39173973 10.1016/j.tjnut.2024.08.012 PMC11600052

[208] Reichardt N, Vollmer M, Holtrop G, Farquharson FM, Wefers D, Bunzel M, et al Specific substrate-driven changes in human faecal microbiota composition contrast with functional redundancy in short-chain fatty acid production. ISME J 2018, 12, 610–22. 29192904 10.1038/ismej.2017.196 PMC5776475

[209] Singh N, Gurav A, Sivaprakasam S, Brady E, Padia R, Shi H, et al Activation of Gpr109a, receptor for niacin and the commensal metabolite butyrate, suppresses colonic inflammation and carcinogenesis. Immunity 2014, 40, 128–39. 24412617 10.1016/j.immuni.2013.12.007 PMC4305274

[210] Gurav A, Sivaprakasam S, Bhutia YD, Boettger T, Singh N, Ganapathy V. Slc5a8, a Na+-coupled high-affinity transporter for short-chain fatty acids, is a conditional tumour suppressor in colon that protects against colitis and colon cancer under low-fibre dietary conditions. Biochem J 2015, 469, 267–78. 25984582 10.1042/BJ20150242 PMC4943859

[211] Chang PV, Hao L, Offermanns S, Medzhitov R. The microbial metabolite butyrate regulates intestinal macrophage function via histone deacetylase inhibition. Proc Natl Acad Sci U S A 2014, 111, 2247–52. 24390544 10.1073/pnas.1322269111 PMC3926023

[212] Park J, Kim M, Kang SG, Jannasch AH, Cooper B, Patterson J, et al Short-chain fatty acids induce both effector and regulatory T cells by suppression of histone deacetylases and regulation of the mTOR-S6K pathway. Mucosal Immunol 2015, 8, 80–93. 24917457 10.1038/mi.2014.44 PMC4263689

[213] Lund PJ, Gates LA, Leboeuf M, Smith SA, Chau L, Lopes M, et al Stable isotope tracing *in vivo* reveals a metabolic bridge linking the microbiota to host histone acetylation. Cell Rep 2022, 41, 111809. 36516747 10.1016/j.celrep.2022.111809 PMC9994635

[214] Zheng Y, Josefowicz S, Chaudhry A, Peng XP, Forbush K, Rudensky AY. Role of conserved non-coding DNA elements in the Foxp3 gene in regulatory T-cell fate. Nature 2010, 463, 808–12. 20072126 10.1038/nature08750 PMC2884187

[215] Collins SL, Stine JG, Bisanz JE, Okafor CD, Patterson AD. Bile acids and the gut microbiota: metabolic interactions and impacts on disease. Nat Rev Microbiol 2023, 21, 236–47. 36253479 10.1038/s41579-022-00805-x PMC12536349

[216] Lee MH, Nuccio SP, Mohanty I, Hagey LR, Dorrestein PC, Chu H, et al How bile acids and the microbiota interact to shape host immunity. Nat Rev Immunol 2024, 24, 798–809. 39009868 10.1038/s41577-024-01057-x

[217] Hang S, Paik D, Yao L, Kim E, Trinath J, Lu J, et al Bile acid metabolites control T(H)17 and T(reg) cell differentiation. Nature 2019, 576, 143–8. 31776512 10.1038/s41586-019-1785-z PMC6949019

[218] Paik D, Yao L, Zhang Y, Bae S, D'Agostino GD, Zhang M, et al Human gut bacteria produce TH17-modulating bile acid metabolites. Nature 2022, 603, 907–12. 35296854 10.1038/s41586-022-04480-z PMC9132548

[219] Li W, Hang S, Fang Y, Bae S, Zhang Y, Zhang M, et al A bacterial bile acid metabolite modulates Treg activity through the nuclear hormone receptor NR4A1. Cell Host Microbe 2021, 29, 1366–77.e9. 34416161 10.1016/j.chom.2021.07.013 PMC9064000

[220] Song X, Sun X, Oh SF, Wu M, Zhang Y, Zheng W, et al Microbial bile acid metabolites modulate gut RORγ(+) regulatory T cell homeostasis. Nature 2020, 577, 410–5. 31875848 10.1038/s41586-019-1865-0 PMC7274525

[221] Campbell C, McKenney PT, Konstantinovsky D, Isaeva OI, Schizas M, Verter J, et al Bacterial metabolism of bile acids promotes generation of peripheral regulatory T cells. Nature 2020, 581, 475–9. 32461639 10.1038/s41586-020-2193-0 PMC7540721

[222] Munn DH, Sharma MD, Baban B, Harding HP, Zhang Y, Ron D, et al GCN2 kinase in T cells mediates proliferative arrest and anergy induction in response to indoleamine 2,3-dioxygenase. Immunity 2005, 22, 633–42. 15894280 10.1016/j.immuni.2005.03.013

[223] Stockinger B, Diaz OE, Wincent E. The influence of AHR on immune and tissue biology. EMBO Mol Med 2024, 16, 2290–8. 39242971 10.1038/s44321-024-00135-w PMC11473696

[224] Ye J, Qiu J, Bostick JW, Ueda A, Schjerven H, Li S, et al The Aryl hydrocarbon receptor preferentially marks and promotes gut regulatory T cells. Cell Rep 2017, 21, 2277–90. 29166616 10.1016/j.celrep.2017.10.114 PMC5880207

[225] Cervantes-Barragan L, Chai JN, Tianero MD, Di Luccia B, Ahern PP, Merriman J, et al *Lactobacillus reuteri* induces gut intraepithelial CD4(+)CD8αα(+) T cells. Science 2017, 357, 806–10. 28775213 10.1126/science.aah5825 PMC5687812

[226] Li Q, de Oliveira Formiga R, Puchois V, Creusot L, Ahmad AH, Amouyal S, et al Microbial metabolite indole-3-propionic acid drives mitochondrial respiration in CD4(+) T cells to confer protection against intestinal inflammation. Nat Metab 2025 2025, 7, 2510–30. 10.1038/s42255-025-01396-6 PMC12727523 41120706

[227] Sanidad KZ, Rager SL, Carrow HC, Ananthanarayanan A, Callaghan R, Hart LR, et al Gut bacteria-derived serotonin promotes immune tolerance in early life. Sci Immunol 2024, 9, eadj4775. 38489352 10.1126/sciimmunol.adj4775 PMC11328322

[228] Blander JM, Medzhitov R. Toll-dependent selection of microbial antigens for presentation by dendritic cells. Nature 2006, 440, 808–12. 16489357 10.1038/nature04596

[229] Remick BC, Gaidt MM, Vance RE. Effector-triggered immunity. Annu Rev Immunol 2023, 41, 453–81. 36750319 10.1146/annurev-immunol-101721-031732

[230] Matzinger P . Tolerance, danger, and the extended family. Annu Rev Immunol 1994, 12, 991–1045. 8011301 10.1146/annurev.iy.12.040194.005015

[231] Hand TW, Dos Santos LM, Bouladoux N, Molloy MJ, Pagán AJ, Pepper M, et al Acute gastrointestinal infection induces long-lived microbiota-specific T cell responses. Science 2012, 337, 1553–6. 22923434 10.1126/science.1220961 PMC3784339

[232] Yang Y, Torchinsky MB, Gobert M, Xiong H, Xu M, Linehan JL, et al Focused specificity of intestinal TH17 cells towards commensal bacterial antigens. Nature 2014, 510, 152–6. 24739972 10.1038/nature13279 PMC4128479

[233] Kullberg MC, Jankovic D, Feng CG, Hue S, Gorelick PL, McKenzie BS, et al IL-23 plays a key role in Helicobacter hepaticus-induced T cell-dependent colitis. J Exp Med 2006, 203, 2485–94. 17030948 10.1084/jem.20061082 PMC2118119

[234] Kullberg MC, Rothfuchs AG, Jankovic D, Caspar P, Wynn TA, Gorelick PL, et al Helicobacter hepaticus-induced colitis in interleukin-10-deficient mice: cytokine requirements for the induction and maintenance of intestinal inflammation. Infect Immun 2001, 69, 4232–41. 11401959 10.1128/IAI.69.7.4232-4241.2001 PMC98456

[235] Kullberg MC, Ward JM, Gorelick PL, Caspar P, Hieny S, Cheever A, et al Helicobacter hepaticus triggers colitis in specific-pathogen-free interleukin-10 (IL-10)-deficient mice through an IL-12- and gamma interferon-dependent mechanism. Infect Immun 1998, 66, 5157–66. 9784517 10.1128/iai.66.11.5157-5166.1998 PMC108643

[236] Morrison PJ, Bending D, Fouser LA, Wright JF, Stockinger B, Cooke A, et al Th17-cell plasticity in Helicobacter hepaticus-induced intestinal inflammation. Mucosal Immunol 2013, 6, 1143–56. 23462910 10.1038/mi.2013.11

[237] Mason D . A very high level of crossreactivity is an essential feature of the T-cell receptor. Immunol Today 1998, 19, 395–404. 9745202 10.1016/s0167-5699(98)01299-7

[238] Kullberg MC, Andersen JF, Gorelick PL, Caspar P, Suerbaum S, Fox JG, et al Induction of colitis by a CD4+ T cell clone specific for a bacterial epitope. Proc Natl Acad Sci U S A 2003, 100, 15830–5. 14673119 10.1073/pnas.2534546100 PMC307653

[239] Perez-Muñoz ME, Joglekar P, Shen YJ, Chang KY, Peterson DA. Identification and phylogeny of the first T cell epitope identified from a human gut bacteroides species. PLoS One 2015, 10, e0144382. 26637014 10.1371/journal.pone.0144382 PMC4670158

[240] Nagashima K, Zhao A, Atabakhsh K, Bae M, Blum JE, Weakley A, et al Mapping the T cell repertoire to a complex gut bacterial community. Nature 2023, 621, 162–70. 37587342 10.1038/s41586-023-06431-8 PMC10948025

[241] Wu S, Rhee KJ, Albesiano E, Rabizadeh S, Wu X, Yen HR, et al A human colonic commensal promotes colon tumorigenesis via activation of T helper type 17 T cell responses. Nat Med 2009, 15, 1016–22. 19701202 10.1038/nm.2015 PMC3034219

[242] Glowacki RWP, Till JM, Brock OD, Engelhart MJ, Ahern PP. Strain-level antigen variation facilitates immune evasion in *Bacteroides thetaiotaomicron*. bioRxiv 629425, 10.1101/2024.12.20.629425, 22 December 2024, preprint: not peer reviewed. PMC13017159 41847847

[243] Kriegel MA, Sefik E, Hill JA, Wu HJ, Benoist C, Mathis D. Naturally transmitted segmented filamentous bacteria segregate with diabetes protection in nonobese diabetic mice. Proc Natl Acad Sci U S A 2011, 108, 11548–53. 21709219 10.1073/pnas.1108924108 PMC3136249

[244] Wu H-J, Ivanov II, Darce J, Hattori K, Shima T, Umesaki Y, et al Gut-residing segmented filamentous bacteria drive autoimmune arthritis via T helper 17 cells. Immunity 2010, 32, 815–27. 20620945 10.1016/j.immuni.2010.06.001 PMC2904693

[245] Lee YK, Menezes JS, Umesaki Y, Mazmanian SK. Proinflammatory T-cell responses to gut microbiota promote experimental autoimmune encephalomyelitis. Proc Natl Acad Sci U S A 2011, 108, 4615–22. 20660719 10.1073/pnas.1000082107 PMC3063590

[246] White Z, Cabrera I, Mei L, Clevenger M, Ochoa-Raya A, Kapustka I, et al Gut inflammation promotes microbiota-specific CD4 T cell-mediated neuroinflammation. Nature 2025, 643, 509–18. 40533562 10.1038/s41586-025-09120-w

[247] Horai R, Zárate-Bladés CR, Dillenburg-Pilla P, Chen J, Kielczewski JL, Silver PB, et al Microbiota-dependent activation of an autoreactive T cell receptor provokes autoimmunity in an immunologically privileged site. Immunity 2015, 43, 343–53. 26287682 10.1016/j.immuni.2015.07.014 PMC4544742

[248] Cebula A, Seweryn M, Rempala GA, Pabla SS, McIndoe RA, Denning TL, et al Thymus-derived regulatory T cells contribute to tolerance to commensal microbiota. Nature 2013, 497, 258–62. 23624374 10.1038/nature12079 PMC3711137

[249] Lee HM, Bautista JL, Scott-Browne J, Mohan JF, Hsieh CS. A broad range of self-reactivity drives thymic regulatory T cell selection to limit responses to self. Immunity 2012, 37, 475–86. 22921379 10.1016/j.immuni.2012.07.009 PMC3456990

[250] Bhattacharjee A, Burr AHP, Overacre-Delgoffe AE, Tometich JT, Yang D, Huckestein BR, et al Environmental enteric dysfunction induces regulatory T cells that inhibit local CD4+ T cell responses and impair oral vaccine efficacy. Immunity 2021, 54, 1745–57.e7. 34348118 10.1016/j.immuni.2021.07.005 PMC8415388

[251] Hegazy AN, West NR, Stubbington MJT, Wendt E, Suijker KIM, Datsi A, et al Circulating and tissue-resident CD4(+) T cells with reactivity to intestinal microbiota are abundant in healthy individuals and function is altered during inflammation. Gastroenterology 2017, 153, 1320–37.e16. 28782508 10.1053/j.gastro.2017.07.047 PMC5687320

